# Synthesis and Structure Elucidation of New NSAID Diphosphine Ruthenium(II) Complexes as Potential Anticancer Agents: DNA/BSA Binding and Cytotoxicity Assays

**DOI:** 10.1021/acsomega.5c12981

**Published:** 2026-04-08

**Authors:** Diogo E. L. Carvalho, Tamara Teixeira, João Honorato de Araujo-Neto, Alzir A. Batista, Keven S. Fragoso, Katia M. Oliveira, Rodrigo S. Corrêa

**Affiliations:** † Department of Chemistry, 28115 Federal University of Ouro Preto (UFOP), Ouro Preto 35402-136, Minas Gerais, Brazil; ‡ Department of Chemistry, Federal University of São Carlos (UFSCar), São Carlos 13561-901, São Paulo, Brazil; § Institute of Chemistry, 28127 University of Brasília (UnB), Campus Darcy Ribeiro, Brasília 70910-900, Distrito Federal, Brazil

## Abstract

New diphosphine ruthenium­(II) complexes with nonsteroidal anti-inflammatory drugs (NSAID) derivatives with general formula [Ru­(L)­(dppe)_2_]­PF_6_ [dppe = 1,2-*bis*(diphenylphosphine)­ethane and (L) corresponds to fenamate (fe^–^) (Complex **1**), mefenamate (me^–^) (Complex **2**), tolfenamate (tol^–^) (Complex **3**) or flufenamate (flu^–^) (Complex **4**)] were synthesized. All compounds were characterized by several techniques, including ^1^H, ^13^C­{^1^H} and ^31^P­{^1^H} NMR, cyclic voltammetry, conductivity, elemental analysis, ultraviolet/visible absorption spectroscopy, infrared absorption spectroscopy and single crystal X-ray diffraction. ct-DNA (Calf-Thymus DNA) interaction studies by spectroscopic titrations and viscosity measurements demonstrated that the complexes may interact weakly with ct-DNA, considering that the complexes are cationic and that DNA carries a negative charge due to its phosphate groups, electrostatic interactions could occur. The complexes showed a moderate interaction with Bovine Serum Albumin (BSA) mediated by both dynamic and static quenching mechanisms. Also, the cytotoxic activity of the complexes was evaluated against breast (MDA-MB-231) and lung (A549) tumor cell lines and nontumor lung (MRC-5) cell lines. All complexes showed cytotoxicity against the cell lines investigated, with IC_50_ values in the range of 0.94–17.71 μM. [Ru­(me)­(dppe)_2_]­PF_6_ showed a high selectivity index against the A549 cell line. For MDA-MB-231 cell line, the most selective complex was [Ru­(fe)­(dppe)_2_]­PF_6_. Except for [Ru­(tol)­(dppe)_2_]­PF_6_, all other complexes were more cytotoxic than cisplatin, making them promising compounds.

## Graphical abstract

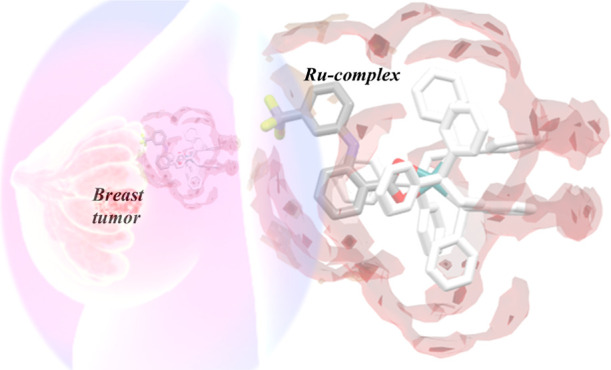

## Introduction

Ruthenium coordination compounds have been extensively investigated as potential alternatives for the diagnosis and treatment of various diseases, including cancer. The significance of these compounds gained prominence following the discovery of the antitumor properties of platinum­(II) complexes, particularly cisplatin, the first metallodrug used in cancer therapy. Cisplatin has proven highly effective in treating testicular cancer and has also demonstrated efficacy against other cancers, such as lung and ovarian cancer.^1–3^ Despite the significant success of cisplatin as a chemotherapeutic agent, its side effects, such as ototoxicity and renal toxicity, along with the development of resistance after prolonged use, have driven the search for new metallodrugs.^4,5^ Consequently, many research groups have focused on discovering new compounds with different metals that are both effective and exhibit fewer side effects. In this context, ruthenium has emerged as a promising alternative.^6^

An effective approach in developing new chemotherapeutic candidates is to use ligands with inherent biological activity.^7,8^ Among these, ligands derived from fenamic acids or *N*-phenylanthranilic acid, which are nonsteroidal anti-inflammatory drugs (NSAIDs), have garnered interest. These NSAIDs work by inhibiting prostaglandin synthesis through the blockade of cyclooxygenase-1 (COX-1) and cyclooxygenase-2 (COX-2), and are commonly used to manage acute or chronic pain associated with inflammatory conditions. Furthermore, ruthenium compounds containing phosphine ligands have been reported as potential candidates for antitumor treatment.^9–12^ Studies demonstrated that ruthenium/phosphine complexes coordinated to diclofenac and ibuprofen (nonsteroidal anti-inflammatory drugs), exhibited significant results against tumor cell lines, making this design promising.^13,14^ Recently, we have demonstrated that Ru­(II)/bipy/dppp complexes with fenamic acid derivatives are promising agents against A2780 and A2780cisR (cisplatin-resistant ovarian tumors).^15^

Therefore, this report aimed to explore the chemical and structural properties of ruthenium complexes containing two diphosphine 1,2-bis­(diphenylphosphino)­ethane (dppe) and one fenamic acid derivative as ligands. Their biological activities against tumor cells have been tuned by changing the fenamate derivative, using fenamate (Complex 1), mefenamate (Complex 2), tolfenamate (Complex 3) or flufenamate (Complex 4). To assess selectivity, the cytotoxicity assays against nontumor cells were carried out. Finally, interaction studies with ct-DNA and bovine serum albumin (BSA) were performed to evaluate aspects related to the mechanism of action and transport of the compounds.

## Experimental Section

The solvents used in the development of this work, such as dichloromethane, methanol, toluene and ethyl ether were acquired from Synth P.A or Merck P.A. The RuCl_3_.*n*H_2_O, 1,2′-*bis*(diphenylphosphino)­ethane (dppe), fenamic acid (fe), mefenamic acid (me), tolfenamic acid (tol) and flufenamic acid (flu), NH_4_PF_6_ = ammonium hexafluorophosphate, Et_3_N = triethylamine, acquired from Aldrich, were used as received.

### Synthesis of the Complex *Trans*-[RuCl_2_(dppe)_2_]

In a round-bottom flash, 1g (4.82 mmol) of RuCl_3_.*n*H_2_O was added to 5 mL of deaerated dimethyl sulfoxide (DMSO). The reaction was refluxed under stirring for approximately 5 h. Subsequently, the system was cooled to room temperature, and acetone (30 mL) was added, forming a yellow-colored precipitate related to *cis*-[RuCl_2_(DMSO)_4_] complex. The precipitate was filtered using a porous plate filter, washed with acetone and ethyl ether, and dried under vacuum (yield: 60%). The complex *trans*-[RuCl_2_(dppe)_2_] was obtained from the reaction of *cis*-[RuCl_2_(DMSO)_4_] with dppe diphosphine. For this, 0.5 g (1.03 mmol) of the *cis*-[RuCl_2_(DMSO)_4_] was added to a Schlenk-type flask containing approximately 20 mL of dichloromethane (DCM), along with 0.94 g (2.37 mmol) of dppe. The reaction was kept under stirring for 5 h and reflux. The volume was reduced and the solid precipitated by the addition of hexane. The pale yellow-colored precipitate was filtered off and washed with hexane and dried under vacuum (yield: 80%).

### Synthesis of the Complexes [Ru­(L)­(dppe)_2_]­PF_6_, L = Fenamate, Mefenamate, Tolfenamate or Flufenamate

The new complexes, [Ru­(L)­(dppe)_2_]­PF_6_, where L are derivate form fenamic, mefenamic, tolfenamic or flufenamic acid, were obtained from the reaction of the precursor *trans*-[RuCl_2_(dppe)_2_] with the ligand of interest in an argon atmosphere. For the synthesis of the complexes with the fenamic acid derivatives, *trans*-[RuCl_2_(dppe)_2_] (0.050 g and 51.6 mmol) was added to a Schlenk flask containing deaerated dichloromethane. In another Schlenk flask containing deaerated methanol, fenamic acid (0.022 g and 103 mmol) was added, together with 30 μL of Et_3_N for the ligand deprotonation. After approximately 30 min of stirring, the solution containing the deprotonated ligand was added to the Schlenk flask containing the solubilized precursor. NH_4_PF_6_ was then added to the reaction mixture, which was kept under reflux. After 1 h, the volume was reduced to approximately 2 mL, and the complex was precipitated with deaerated distilled water. The product formed was collected by filtration, washed with water, ether and dried under vacuum. After drying, the solid was recrystallized by slow evaporation of a methanol/dichloromethane solvent mixture (1:1, v/v). The same procedure was used for complexes containing mefenamic acid ([Ru­(me)­(dppe)_2_]­PF_6_), flufenamic acid ([Ru­(flu)­(dppe)_2_]­PF_6_) and tolfenamic acid ([Ru­(tol)­(dppe)_2_]­PF_6_).

### [Ru­(fe)­(dppe)_2_]­PF_6_ (**1**)

Yield: 37 mg (57%). Color: Pale yellow. Elementary Analysis (%) for (RuC_65_H_58_F_6_NO_2_P_5_): Calculated: C, 62.15; H, 4.62; N, 1.12; Found: C, 62.02; H, 5.12; N, 1.25. Selected IR (cm^–1^): 3329, 2931, 1597, 1582, 1435, 1288, 1097, 832, 685, 557. UV–vis (methanol, 1.0 × 10^–5^ M): λ/nm (ε/M^–1^cm^–1^) 258 (30802), 290 (10741), 364 (7400). Molar conductivity (ΛM, S.m^2^.mol^–1^) = 97. Decomposition temperature (°C): >300. ^31^P­{^1^H} NMR (CDCl_3_, 400 MHz) δ/ppm: 58.78 (P1, P2), 57.76 (P3, P4), −144 (hept, ^3^JP-P = 19.6 Hz, PF_6_^–^). ^1^H NMR (400 MHz, CDCl_3_) δ: (ppm): 8.99 (N–H); 3.01–2.26 (dppe-H); 7.82–6.91 (Ar-dppe-H); 8.99 (N–H). ^13^C­{^1^H} NMR (CDCl_3_, 100 MHz) δ: (ppm): 182.03 (C–O); 145.05 × 10^140.93^ (C–N); 132.67–133.44 and 130.74–131.67 (C-PPh_2_); 128.91–128.72 and 128.42–128.09 (C-dppe).

### [Ru­(me)­(dppe)_2_]­PF_6_ (**2**)

Yield: 60 mg (90%). Color: Pale yellow. Elementary Analysis (%) for (RuC_67_H_62_F_6_NO_2_P_5_): Calculated: C, 62.66; H, 4.83; N, 1.11; Found: C, 62.88; H, 4.30; N, 1.30%. Selected IR (cm^–1^): 3325, 3062, 1616, 1581, 1435, 1288, 1092, 833, 690, 555. UV–vis (methanol, 1.0 × 10^–5^ M: λ/nm (ε/M^–1^cm^–1^) 258 (55728), 364 (13431). Molar conductivity (ΛM, S.m^2^.mol^–1^) = 146. Decomposition temperature (°C): 295. ^31^P­{^1^H} NMR (CDCl_3_, 400 MHz) δ/ppm: 58.82 (P1, P2), 56.97 (P3, P4), −144 (hept, ^3^JP-P = 19.6 Hz, PF_6_^–^). ^1^H NMR (400 MHz, CDCl_3_) δ: (ppm): 3.01–2.26 (dppe-H); 7.82–6.93 (Ar-dppe-H); 8.62 (N–H). ^13^C­{^1^H} NMR (CDCl_3_, 100 MHz) δ: (ppm): (125.74 MHz, CDCl3) δ: (ppm): 182.46 (C–O); 146.93 × 10^138^.80 (C–N); 133.46–129.64 and 132.73–131.88 (C-PPh_2_); 128.90–128.70 and 128.33–128.14 (C-dppe); 20.80 and 14.40 (C–H).

### [Ru­(tol)­(dppe)_2_]­PF_6_ (**3**)

Yield: 34 mg (50%). Color: Pale yellow. Elementary Analysis (%) for (RuC_66_H_59_ClF_6_NO_2_P_5_): Calculated: C, 60.76; H, 4.53; N, 1.07; Found: C, 61.12; H, 4.18; N, 1.64. Selected IR (cm^–1^): 3307, 2931, 1614, 1583, 1433, 1288, 1089, 831, 689, 555. UV–vis (methanol, 1.0 × 10^–5^ M: λ/nm (ε/M^–1^cm^–1^): λ/nm (ε/M^–1^cm^–1^) 258 (45606), 290 (13836), 364 (11436). Molar conductivity (ΛM, S.m^2^ mol^–1^) = 96. Decomposition temperature (°C): 297. ^31^P­{^1^H} NMR (CDCl_3_, 400 MHz) δ/ppm: 58.90 (P1, P2), 57.11 (P3, P4), −144 (hept, ^3^JP-P = 19.6 Hz, PF_6_^–^). ^1^H (400 MHz, CDCl_3_) δ: (ppm): 2.93–2.27 (dppe-H); 7.95–6.42 (Ar-dppe-H); 8.58 (N–H). ^13^C­{^1^H} NMR (CDCl_3_, 100 MHz) δ: (ppm): 182.07 (C–O); 146.68 and 140.60 (C–N); 135.58 (C–Cl); 132.38 and 130.70 (C-PPh2); 128.95–128.15 and 126.96–126.90 (C-dppe); 15.43 (C–H).

### [Ru­(flu)­(dppe)_2_]­PF_6_ (**4**)

Yield: 65 mg (95%). Color: Pale yellow. Elementary Analysis (%) for (RuC_66_H_57_F_9_NO_2_P_5_): Calculated: C, 59.86; H, 4.31; N, 1.06; Found: C, 60.01; H, 3.95; N, 1.22. Selected IR (cm^–1^): 3321, 3056, 1610, 1589, 1435, 1346, 1325, 1294, 1219, 1130, 1095, 831, 557, 688. UV–vis (methanol, 1.0 × 10^–5^ M): λ/nm (ε/M^–1^cm^–1^) 258 (36424), 290 (17609), 364 (9461). Molar conductivity (ΛM, S.m^2^.mol^–1^) = 94. Decomposition temperature (°C): 265. ^31^P­{^1^H} NMR (CDCl_3_, 400 MHz) δ/ppm: 59.05 (P1, P2), 57.90 (P3, P4), −144 (hept, ^3^JP-P = 19.6 Hz, PF_6_^–^). ^1^H NMR (400 MHz, CDCl_3_) δ: (ppm): 3.02–2.26 (dppe-H); 7.82–6.06 (Ar-dppe-H, flu-H); 9.12 (N–H). ^13^C­{^1^H} NMR (CDCl_3_, 100 MHz) δ: (ppm): 181.52 (C–O); 143.64 and 141.86 (C–N); 131.07 and 129.62 (C-PPh_2_); 132.86–128.74 and 128.43 (C-dppe); 122.74 (C–F).

### Physical Measurements

The conductivity measurements of the complexes were performed using a conductivity meter (Meter Lab., model CDM230) on solutions of complexes at a concentration of 1 × 10^–3^ mol L^–1^, in methanol at room temperature (23 °C). For the measurements of the melting point of the complexes, a Fisatom 431 D equipment was used. The elemental analyzes (% C, H and N) were performed using a PerkinElmer 2400 CHNS Analyzer. The IR spectra of the synthesized complexes and the free ligands were obtained in the region from 4000 to 400 cm-1, using an ABB Bomem MB3000 Spectrometer (Quebec, Canada). Electrochemical studies of ruthenium­(II) complexes were performed using the cyclic voltammetry technique. Analyzes were performed at room temperature using an Autolab Potentiostat/Galvanostat, model PGSTAT302N. The measurements were performed in a conventional three-electrode electrochemical cell: a reference electrode Ag/AgCl (KCl 3.0 mol L^–1^) and working and auxiliary platinum electrodes, dipped in PTBA (tetrabutylammonium perchlorate) electrolyte solution (0.1 mol L^–1^ in CH_2_Cl_2_). Ultraviolet–visible (UV–vis) absorption spectra were obtained in the range of 200 to 800 nm, with a Thermo Scientific-Genesys 10s UV/vis spectrophotometer, using quartz cuvettes with a 1 cm optical path at room temperature. The stability studies of the ruthenium compounds were carried out using the UV–vis absorption spectroscopy technique in DMSO and in a mixture of DMSO and *Tris*–HCl Buffer (pH 7.4) 50:50 (v/v) for a period of 0 to 72 h.

^1^H, ^13^C­{^1^H} and ^31^P­{^1^H} spectra were obtained using a Bruker Avance 400 MHz spectrometer. The ^1^H and ^13^C­{^1^H} NMR experiments used the solvent deuterated chloroform (CDCl_3_). In order to obtain the ^31^P­{^1^H} RMN spectra, the complexes were solubilized in CH_2_Cl_2_ and added to the NMR tubes together with a D_2_O capillary. All spectra were obtained at room temperature.

The single crystals obtained in this work were obtained by slow evaporation of the methanol/dichloromethane solvent mixture (1:1 v/v). X-ray diffraction measurements were performed in a Rigaku XtaLAB mini II diffractometer, with graphite monochromated Mo Kα radiation (λ = 0.71073 Å). Diffraction data were collected at room temperature and treated by specific crystallographic software. The WINGX^16^ software package was used in the data analysis. The structures were solved using direct methods, with the SHELXS-97 software.^17^ Thus, the models obtained were refined (full matrix least-squares) in F2 using the SHELXL-97 software. The Mercury 4.0 ^18^ software was used to analyze and create graphical representations of the structures.

### Evaluation of ct-DNA Interactions of the Complexes **1**-**4**

To investigate the interaction of the obtained ruthenium complexes with ct-DNA, spectroscopic titrations by UV–vis and viscosity measurements were used. Initially, the ct-DNA solution was prepared by solubilizing approximately 2 mg of ct-DNA (*Calf Thymus*) in 1 mL of *Tris*–HCl buffer (4.5 mmol L^–1^ of *Tris* HCl, 0.5 mmol L^–1^ of *Tris*-base, and 50 mmol L^–1^ of NaCl, pH 7.4). The concentration of ct-DNA was determined by UV–vis spectroscopy, using the absorbance value at 260 nm and the molar absorptivity of 6600 mol^–1^ cm^–1^ L, according to Lambert–Beer law (A_260_ = ε_260_ × b × c). To perform the UV–vis spectroscopic titrations, a solution of each complex was prepared at a concentration of 1.5 × 10^–3^ molL^–1^ in DMSO. From this stock solution, a new solution of the complex in *Tris*–HCl buffer, containing 10% DMSO, was prepared. The UV–vis spectrum of the complex was then recorded. Subsequently, 10 μL aliquots of ct-DNA were added to both the blank cuvette and the complex cuvette to ensure that any spectral variation observed was due to the interaction with the ct-DNA. The solutions were homogenized for 1 min and the spectra recorded. The binding constant (*K*_b_) between the complexes and ct-DNA was determined using the following eq 1.^19^

1 $$\frac{\left[DNA\right]}{\left(\varepsilon_{a}-\varepsilon_{f}\right)}=\frac{\left[DNA\right]}{\left(\varepsilon_{b}-\varepsilon_{f}\right)}+\frac{1}{\left[K_{b}\left(\varepsilon_{b}-\varepsilon_{f}\right)\right]}$$

where: ε_a_ = is the apparent extinction coefficient, which corresponds to the ratio between the measured absorbance and the concentration of the complex (A_observed_/[Complex]); ε_f_ = molar absorptivity of the free complex (no addition of ct-DNA); ε_b_ = molar absorptivity of the ct-DNA-bound complex; *K*_b_ = binding constant. From the graph of [DNA]/ (ε_a_–ε_f_) versus [DNA], a straight line with slope of 1/ (ε_b_–ε_f_) and intercept of 1/*K*_b_ (ε_b_–ε_f_) is obtained. Thus, *K*_b_ is determined from the ratio between the slope (angular coefficient) and the intercept (linear coefficient). It is worth to mention that the fraction of bound complex remained limited, resulting in *r* (ratio of bound complex to DNA base pairs) values below 0.1. Therefore, the condition r near 0 required for the valid application of the Wolfe-Shimer equation can be considered satisfactorily fulfilled under our experimental conditions.

The viscosity analysis was conducted using an Ostwald viscometer placed in a water bath at a temperature of 37 °C. The concentration of the ct-DNA solution in *Tris*–HCl buffer was fixed at 50 μmol L^–1^, while the concentration of complexes solubilized in DMSO (with the final percentage of DMSO remaining at 2% in all solutions) varied to achieve different complex/ct-DNA ratios. The measurement time, performed in five replicates, was recorded using a digital stopwatch. The specific viscosity values (η/η_0_)^1/3^, where η corresponds to the relative viscosity of ct-DNA in the presence of complexes and η_0_ is the relative viscosity of ct-DNA, were plotted versus [complex]/[DNA].

### Interaction with BSA of Ru Complexes

The interaction evaluation of the ruthenium complexes with BSA relied on measuring the fluorescence suppression of tryptophan residues of BSA. 2.5 μM of BSA solution was prepared in *Tris*–HCl buffer (4.5 mmol L^–1^ *Tris* HCl, 0.5 mmol L^–1^ *Tris* base and 50 mmol L^–1^ NaCl) at a pH of 7.4. The concentration of BSA was determined using the molar absorptivity of BSA at 279 nm (43824 mol^–1^Lcm^–1^). BSA solutions containing different concentrations of the complexes (0–12.5 μM) were prepared, maintaining a fixed percentage of DMSO at 10%. Subsequently, 200 μL of these solutions were added to opaque 96-well plates (Costar) in triplicate. Measurements were conducted using a SpectraMax i3 fluorimeter at temperatures of 25 and 37 °C, with excitation at 280 nm and emission at 340 nm.

### Ruthenium Complexes Cytotoxic Activity against Breast and Lung Tumor Cell Lines

In order to investigate the cytotoxic activity of the ruthenium complexes, the MDA-MB-231 triple negative breast cancer cell line, the A549 lung cancer cell line, and the nontumor lung cell line MRC-5 were utilized. The cell lines MDA-MB-231 (ATCC: HTB-26), A549 (ATCC: CCL-185), MRC-5 (ATCC: CCL-171) were cultured in Dulbecco’s Modified Eagle Medium (DMEM) supplemented with 10% fetal bovine serum (FBS), penicillin (100 IU/ml), streptomycin (100 mg/mL) and l-glutamine (2 mM). The cells were maintained in a humidified incubator with 5% CO_2_ at a constant temperature of 37 °C in culture flasks.

For the experimentation, the cells were trypsinized, counted to adjust the cell concentration, and then seeded into 96-well culture plates (Corning Costar) at a density of 1.5 × 10^4^ cells/well. The plates were placed in an incubator (37 °C - 5% CO_2_) for 24 h to allow for cell adhesion. Following this incubation period, the ruthenium complexes were added at different concentrations (100–0.78 μM), and the plates were returned to the incubator for 48 h. The percentage of DMSO used in the experiment was 0.5%, with control wells receiving the same percentage of DMSO. After 48 h, the culture medium was removed and 50 μL of MTT (0.5 mg/mL in PBS) was added to each well. The plates were then incubated in the incubator (37 °C–5% CO_2_) for 3–4 h. During this incubation, MTT is reduced by mitochondrial dehydrogenase in viable cells, forming violet-colored formazan crystals. The formazan crystals were solubilized by the addition of isopropanol, and the absorbance of the wells was measured using a microplate reader (Labtech LT4000) at a wavelength of 540 nm. From the absorbance values, the IC_50_ values (the concentration of complex that inhibits 50% of cell growth) were determined.

## Results and Discussion

### Synthesis and Characterization

The new complexes containing fenamic derivatives as ligands were obtained by replacing the chlorido ligands of the precursor complex [RuCl_2_(dppe)_2_], according to the synthesis route shown in Figure 1. An interesting aspect to note is that the precursor complex [RuCl_2_(dppe)_2_] may exist in cis and trans isomers. Usually, the complexes obtained from the precursor [RuCl_2_(dppe)_2_] originate from the cis isomer,^20,21^ as the chloride ligand is more labile when it is trans to phosphorus, resulting in a faster ligand exchange. However, the formation of the *cis* isomer occurs from the *trans* isomer, and the yield is not particularly high. Therefore, here, the synthesis procedure was carried out starting from *trans*-[RuCl_2_(dppe)_2_] under reflux conditions. As the precursor isomerizes to the *cis*-[RuCl_2_(dppe)_2_] species, the reaction initiates, resulting in the formation of the *tris*-chelate complexes [Ru­(L)­(dppe)_2_]­PF_6_, thus naturally presenting the ligands in the *cis* configuration. The chemistry of complexes based on the [Ru­(L)­(dppe)_2_]^+^ core coordinated to bidentate carboxylate or other bioactive ligands has been well documented in the literature.^20,21^ For example, mononuclear ruthenium­(II) complexes of the general formula [Ru­(η^2^-O_2_CR)­(dppe)_2_]­PF_6_ have been synthesized and structurally characterized from thiophene-carboxylate,^20^ ^,^ illustrating the versatility of the {Ru­(dppe)_2_} moiety, forming stable carboxylate chelates, with many applications.

**Figure 1:**
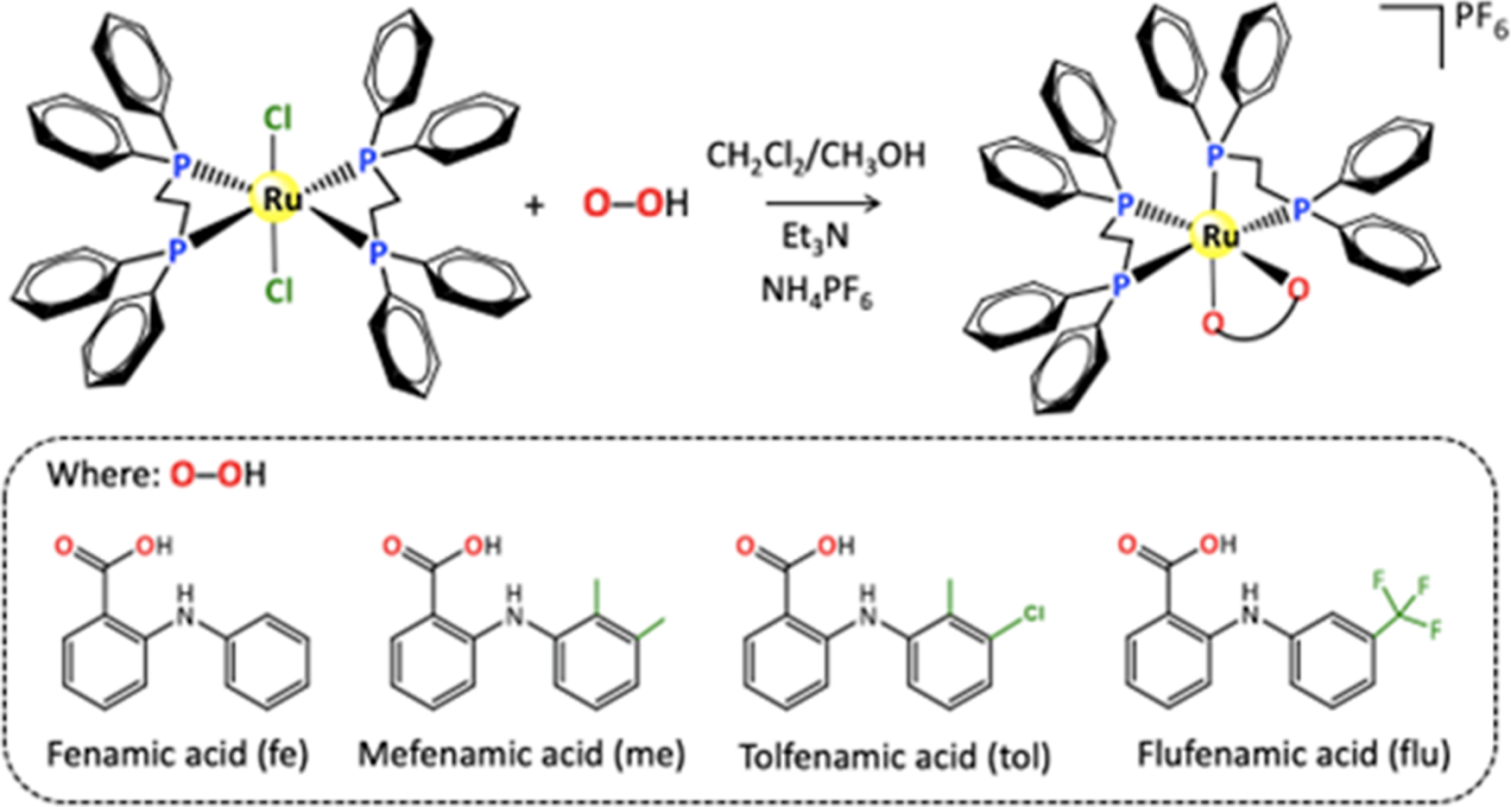
Synthetic route used to obtain complexes containing fenamic acid and derivatives.

During the synthesis, the complexes [Ru­(fe)­(dppe)_2_]­PF_6_, [Ru­(me)­(dppe)_2_]­PF_6_, [Ru­(tol)­(dppe)_2_]­PF_6_, and [Ru­(flu)­(dppe)_2_]­PF_6_ were obtained with respective yields of 57, 91, 51 and 95%. The complexes obtained are pale yellow solids, soluble in organic solvents such as methanol, ethanol, dichloromethane, acetone, chloroform and DMSO. On the other hand, they are insoluble in hydrocarbons, ether and water. The compounds are nonhygroscopic, stable in air and exhibit high thermal stability, indicated by the relatively high decomposition points above 250 °C. Conductivity measurements ranged from 75 to 146 S cm^2^ mol^–1^, indicating that the compounds are 1:1 electrolytes, with the complex acting as a cation and PF_6_^–^ as an anion, according to standards reported in the literature.^22^

The infrared absorption spectroscopy technique confirmed the ligand coordination. Fenamic derivative ligands, which can coordinate via the secondary amine as well as a carboxylate group. Infrared spectra show that both in free ligands and the complexes present a band in the region of 3055–3329 cm^–1^ attributed to the v­(N–H) stretching of the amine. The observed results indicate that metal coordination did not occur through the amine group, suggesting that coordination may be associated with the carboxylate group. This is further supported by the shift in the symmetric and asymmetric stretching bands characteristic of the carboxylate group, specifically v_s_COO^–^ and v_as_COO^–^. In the free ligands, these bands are located in the region of 1648–1431 cm^–1^, whereas in the metal complexes, they are observed at 1597 and 1433 cm^–1^, respectively. The coordination mode can be distinguished from the difference in wavenumbers between v_s_COO^–^ and v_as_COO^–^ (Δcm^–1^). Coordination via the carboxylate group in a monodentate mode typically results in a larger Δvalue compared to the free ligand. On the other hand, bidentate coordination is associated with a smaller Δvalue. Additionally, the v_as_COO^–^ stretch is known to shift to higher frequencies in the monodentate coordination, while a shift to lower frequencies indicates bidentate coordination. For the ruthenium complexes studied, the v_as_COO^–^ stretching frequency is observed to shift to lower wavenumbers, and the Δvalues are smaller compared to those of the free ligands. These observations may suggest that the carboxylate group coordinates to ruthenium in a bidentate manner. Furthermore, the infrared spectra of the complexes showed bands in the region of 831 v­(P–F) and 555 δ­(P–F) cm^–1^, confirming the presence of the PF_6_^–^ anion in the complexes.^23^ In general, all complexes exhibited similar behavior (Figures S1–S4).

The precursor complex *trans*-[RuCl_2_(dppe)_2_] has an oxidation potential of RuII/RuIII at 0.64 V. The new complexes containing fenamic acid and its derivatives showed RuII/RuIII oxidation processes in the region of 1.26 to 1.57 V, which are more positive compared to the precursor complex. This observation is due to the replacement of two chlorido ligands, because they are good σ- and π-electron donors, by carboxylate oxygens of the fenamic ligands and derivatives that are not good electron density donors due to the resonance of the (O–C–O)- group.

The electrochemical studies show the anodic peak potential (*E*_pa_) around of 1.5 V. It was found that the compound with the lowest RuII/RuIII oxidation potential value is the one with mefenamic ligand ([Ru­(me)­(dppe)_2_]­PF_6_), because mefenamic ligand has two methyl groups, which act as electron density donors for one of its benzene rings. Thus, this is the ligand with the greatest basicity. In the case of the complex containing fenamic acid, which does not have substituents on the rings, the RuII/RuIII oxidation potential is 1.31 V, therefore it is greater than that recorded for the complex containing mefenamic acid (1.26 V). When analyzing the complexes containing halogens as substituents, which withdraws electron density ([Ru­(tol)­(dppe)_2_]­PF_6_ and [Ru­(flu)­(dppe)_2_]­PF_6_), the RuII/RuIII oxidation potential increase from the complex with tolfenamic ligand (1.46 V) to the complex with flufenamic acid (1.55 V). The former one has three fluoride substituents in its structure, making it the most acidic among those studied ligands. The potential increasement is less pronounced because the fluorine atoms are not directly attached to the ring. The greater acidity of flufenamic acid (p*K*_a_ = 3.97) compared to other ligands (p*K*_a_ > 4)^24^ justifies the observed electrochemical behavior.

The electronic spectra of fenamic-derived ligands and ruthenium complexes were obtained in a methanol solution to determine the electronic transitions present and their respective molar absorptivities (ε). The spectra of the complexes (Figure S5–S8) showed intraligand π → π* transition bands at 257–258 nm for dppe and at 280–290 nm for the fenamic ligands. A broad band can also be seen in the 354–364 nm region, probably caused by a mixture of *n*→π* transitions around the secondary amine of the fenamic ligands and metal–ligand charge transfer (MLCT). Additionally, the UV–vis absorption spectra of the complexes were obtained in DMSO, and also in a mixture of DMSO and *Tris*–HCl buffer (pH 7.4) at a 50:50 (v/v) ration for a period of 0 to 72 h. No changes or the appearance of new bands were observed in the spectra obtained for the complexes, leading to the conclusion that the compounds are stable under both conditions.

The ^31^P­{^1^H} NMR technique was used to confirm the synthesis of the new Ru­(II) complexes. Initially, the ^31^P­{^1^H} NMR spectrum of the precursor complex *trans*-[RuCl_2_(dppe)_2_] shows a singlet at δ: 43 ppm, which is a characteristic of phosphines in trans positions to each other. Upon performing the syntheses, it was observed that the ^31^P­{^1^H} NMR data obtained for the new complexes are different compared to the chemical shift of the precursor *trans*-[RuCl_2_(dppe)_2_], as well as the cis isomer, indicating the formation of new species. Analyzing the ^31^P­{^1^H} NMR spectrum of the [Ru­(fe)­(dppe)_2_]­PF_6_ complex (Figure S18), a signal was noted around δ: 57 ppm and a broad singlet at δ: 58 ppm, both of which are at higher frequencies relative to the precursor. This shift may be attributed to the weaker electron-donating character of the oxygen atoms in the carboxylate group. In contrast, chlorido ligands, which are stronger donors in terms of σ and π interactions, enrich the electron density at the metal center, thus shifting the signal of the phosphorus atom to higher frequency regions. The ^31^P­{^1^H} NMR spectra of the complexes [Ru­(me)­(dppe)_2_]­PF_6_, [Ru­(tol)­(dppe)_2_]­PF_6_, and [Ru­(flu)­(dppe)_2_]­PF_6_ exhibited similar behavior.

The ^1^H NMR spectra of the complexes (Figure S10–S13) showed two distinct regions identified as one containing the aliphatic hydrogens of the dppe (3–2 ppm), and the other containing the aromatic hydrogens of dppe and the fenamic ligands (8–6 ppm). In the spectra of the free ligands, a signal corresponding to the hydrogen of the O–H group is observed around 11.5 ppm, while in the spectra of the complexes, this signal was not detected, indicating deprotonation and coordination by the carboxylate group. This coordination can also be observed in the chemical shift of the carboxylate carbon in the ^13^C­{^1^H} NMR spectra (Figures S14–S17). In the spectrum of the free ligand, the chemical shift of the carbon from the carboxylate carbon is observed at around 173 ppm, whereas after coordination with ruthenium, this signal shifted to 182 ppm. This shift relative to the free ligand is due to electronic delocalization in the carboxylate ion after its coordination to ruthenium.

The crystal structures of the complexes were determined by single-crystal X-ray diffraction (Figure 2), confirming the proposed structures. The complexes exhibit a distorted octahedral geometry, as evidenced by the bond angles. In all structures, the ligand coordinates in a bidentate mode through the oxygen atoms of the carboxylate group, forming a four-membered ring. The bond angles of less than 90° indicate a distortion from the ideal octahedral geometry. Additionally, the formation of five-membered chelate rings is observed due to the bidentate coordination of the dppe ligand, with bond angles around 83° in the P1–Ru–P2 and P3–Ru–P4 bonds.

**Figure 2:**
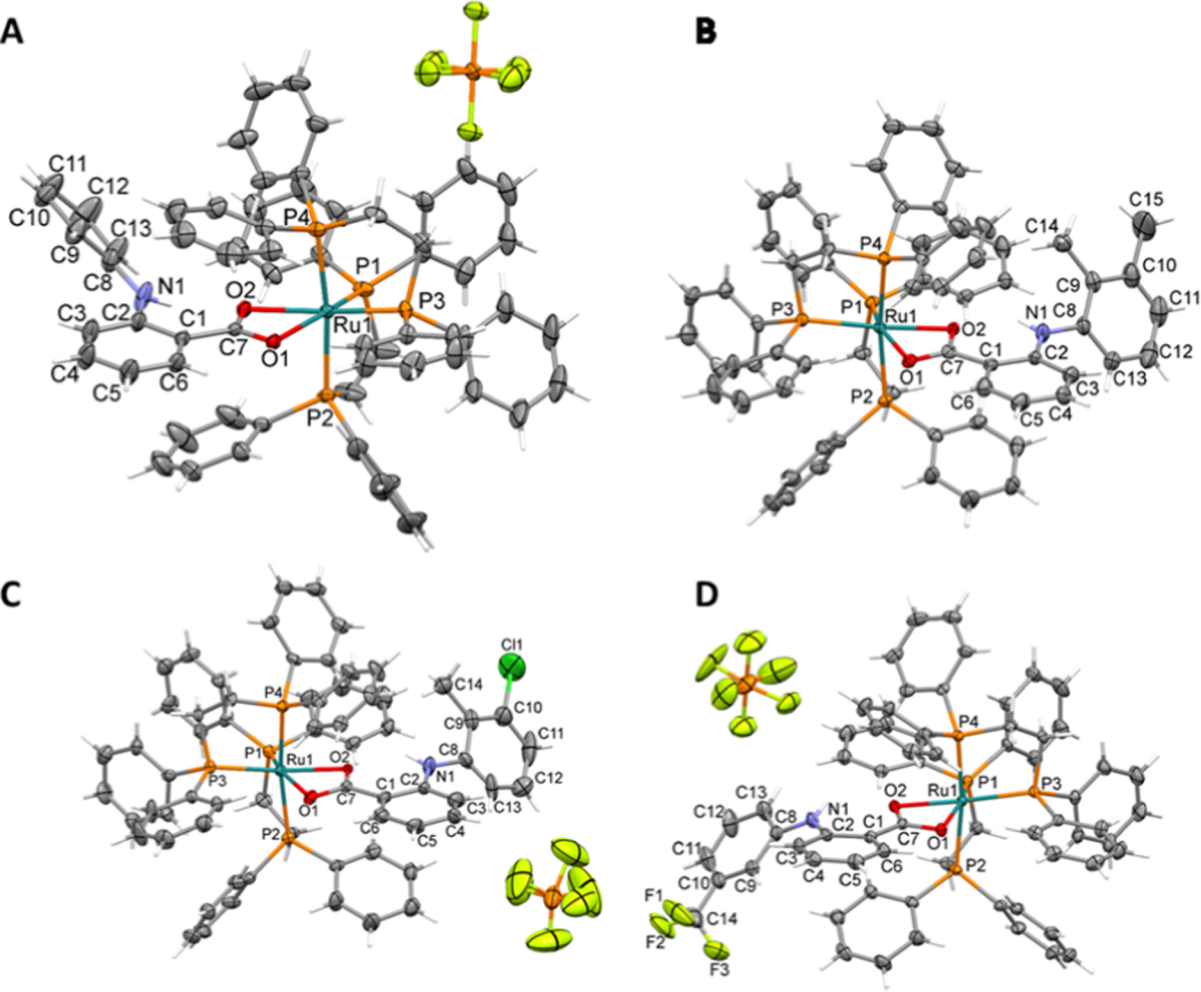
Crystal structures of [Ru­(fe)­(dppe)_2_]­PF_6_ (A), [Ru­(me)­(dppe)_2_]­PF_6_ (B), [Ru­(tol)­(dppe)_2_]­PF_6_ (C) and [Ru­(flu)­(dppe)_2_]­PF_6_ (D) complexes with the ellipsoids represented at 30% probability. Counterion of structure (B) was omitted to clarity.

### Interaction of the Complexes **1**-**4** with Ct-DNA

Elucidating the interactions between metal complexes and DNA is crucial for understanding their biological activity, and a well-known example is cisplatin, whose antitumor effect may be due to its ability to bind to the nitrogenous bases of DNA, blocking replication and leading to cell death.^25^

Consequently, the investigation of ct-DNA binding has become a common approach in the evaluation of potential anticancer agents. Interactions between metal complexes and ct-DNA are generally classified as covalent or noncovalent, the latter including intercalative, groove-binding, and electrostatic modes. UV–vis spectroscopic titration is a straightforward technique commonly used to assess the interaction of compounds with ct-DNA. This method involves monitoring the specific absorption bands of the compound in the presence of varying ct-DNA concentrations. Data analysis allows for the determination of the binding constant (*K*_b_), which indicates the affinity between the compound and ct-DNA. In the present study, the interaction of complexes **1**-**4** with ct-DNA was evaluated by following changes in their characteristic absorption band centered at 262 nm, attributed mainly to ligand-centered π–π* transitions.

Figure 3 shows the UV–vis absorption spectrum for the [Ru­(me)­(dppe)_2_]­PF_6_ complex after successive additions of ct-DNA aliquots. As the concentration of ct-DNA in the solution increases, there is a decrease in the intensity of the absorption bands of the complex, indicating hypochromism. This is a result of the energy variations involved in the electronic transition due to the interactions between the complex and ct-DNA.^26^ It is noteworthy that all complexes showed the same behavior.

**Figure 3:**
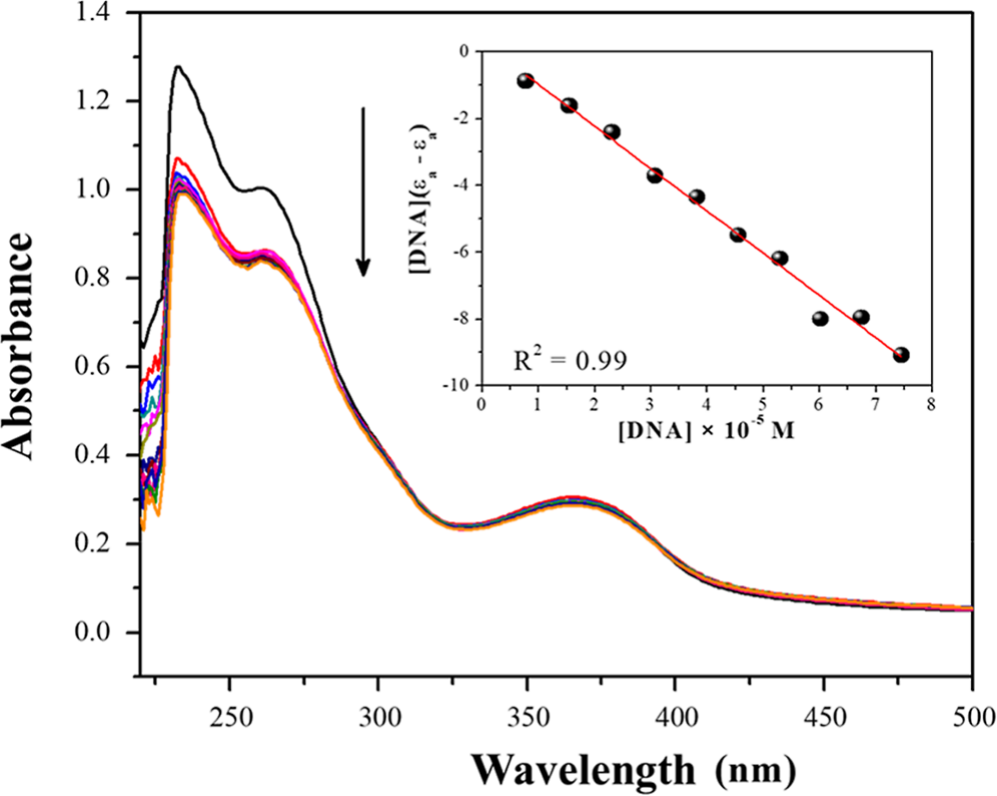
UV–vis absorption spectra of the [Ru­(me)­(dppe)_2_]­PF_6_ (**2**) in the presence of different concentrations of ct-DNA. Inserted graph: [DNA]/ (ε_a_–ε_f_) versus [DNA].

The values for the *K*_b_ constants were determined from the ratio between the angular and linear coefficient of the straight lines obtained by the graphs of [DNA]/ (ε_a_–ε_f_) versus [DNA], and the results obtained are shown in Table 1, together with the percentage of hypochromism. The complexes presented *K*_b_ values with magnitudes of 10^4^–10^5^ M^–1^, which indicate moderate interaction with ct-DNA by an electrostatic interactions and nonintercalative way, when compared to other complexes reported in the literature.^27^

**Table 1:** Binding constants (*K*_b_) and hipocromism (% H) values for complexes **1-4** interacting with ct-DNA, determined by UV–vis spectroscopic titrations

complex	*K*_b_ (L mol^–1^)	λ (nm)	% H
**(1)**	5.5 ± 0.2 × 10^4^	263	13.0
	1.2 ± 0.3 × 10^4^	350	3.5
**(2)**	4.2 ± 0.1 × 10^5^	262	3.6
	8.2 ± 0.1 × 10^4^	350	6.0
**(3)**	4.9 ± 0.2 × 10^5^	262	15.0
	7.0 ± 0.8 × 10^4^	358	3.0
**(4)**	3.6 ± 0.5 × 10^4^	262	17.0
	0.3 ± 0.2 × 10^4^	357	4.5

Viscosity measurements provide valuable insights into the modes of interaction between small molecules and ct-DNA. Compounds that bind covalently to ct-DNA, such as cisplatin, typically induce a decrease in solution viscosity as a result of shortening of the ct-DNA double helix. In contrast, classical intercalators cause an increase in viscosity due to the elongation of the ct-DNA duplex upon insertion between base pairs. Conversely, molecules that interact through groove or by electrostatic forces usually do not produce significant structural perturbations in the ct-DNA, and therefore minimal effects on the viscosity may be observed.^28^ To gain more insight into the type of interaction between the ruthenium complexes and ct-DNA, a viscosity test was conducted. Figure 4 illustrates the plot of (η/η_0_)^1/3^ versus [compound]/[DNA], where η and η_0_ represent the specific viscosities of ct-DNA solutions in the presence and absence of the compounds, respectively, including ruthenium complexes and thiazole orange, a classic intercalator used for comparison.^29^ In the presence of the complexes, the viscosity of the ct-DNA did not change significantly as the [complex]/[DNA] ratio increased, which is not consistent with covalent binding and classical intercalative interactions.

**Figure 4:**
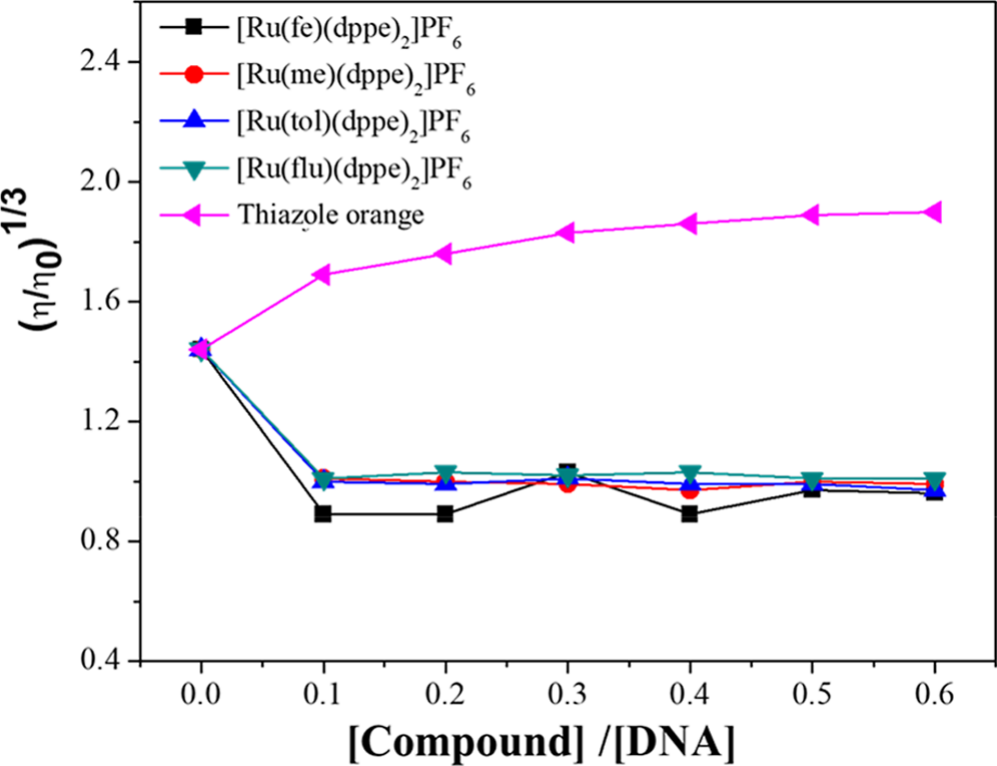
Effect of Ru­(II) complexes and thiazole orange on the relative viscosity of ct-DNA.

Taken together, the observed spectroscopic hypochromism combined with the absence of significant changes in viscosity suggests that the ruthenium complexes interact with ct-DNA through a nonclassical binding mode, possibly involving electrostatic interactions, given that the complexes are cationic and DNA carries a negative charge.

### Interaction of Complexes **1**-**4** with BSA

Human serum albumin (HSA) is the most abundant protein in blood plasma and plays a central role in the transport and distribution of endogenous and exogenous molecules, including therapeutic agents. Therefore, assessing the interaction of prospective metallodrugs with this protein is essential for understanding their pharmacokinetic behavior.^30^ In this study, bovine serum albumin (BSA) was employed as a model protein due to its close structural homology to HSA and its greater availability.^31^ Fluorescence spectroscopy is widely employed for this purpose, considering that BSA exhibits high fluorescence when excited at 280 nm, primarily due to the presence of tryptophan residues in its structure.^32^ Hence, the fluorescence emission spectra of BSA (2.5 μM) in the absence and presence of varying concentrations of ruthenium complexes (0–12.5 μM) were recorded after excitation at 280 nm, with emission measured at 340 nm. As shown in Figure 5, with increasing concentration of the [Ru­(me)­(dppe)_2_]­PF_6_ complex, there was a significant decrease in BSA fluorescence intensity, indicating interaction of the complex with the protein.

**Figure 5:**
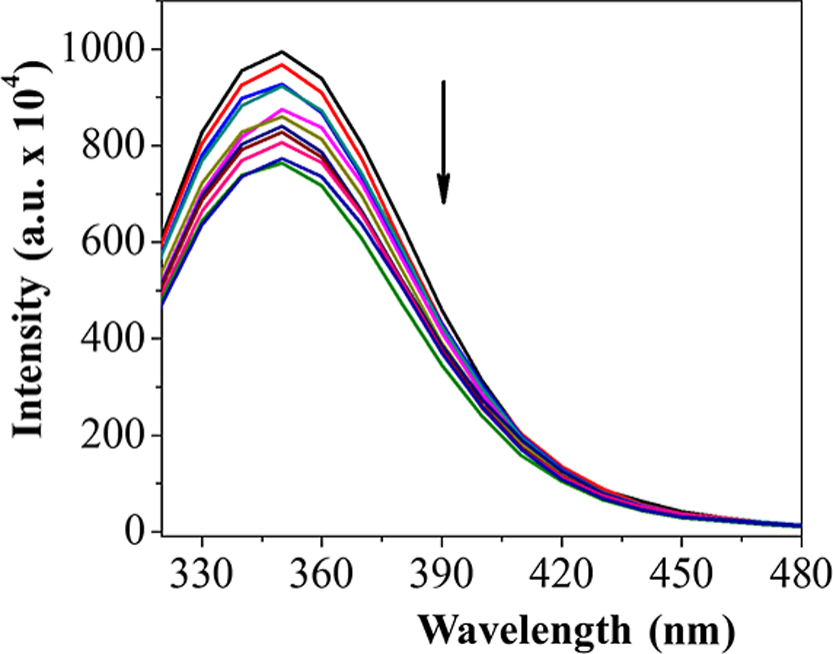
BSA emission spectrum (2.5 μM, λ_ex_ = 280 nm) at different concentrations of the complex [Ru­(me)­(dppe)_2_]­PF_6_ (0–12.5 μM), at 37 °C.

The interaction of complexes with Bovine Serum Albumin (BSA) can occur through two main mechanisms: dynamic and static. To determine which mechanism is active, the experiment was conducted at two different temperatures, 298 and 310 K. Typically, an increase in temperature leads to a higher suppression constant for the dynamic mechanism, while the static mechanism usually results in a lower suppression constant.^31^

The suppression constant (*K*_sv_) was determined by treating the data using the Stern–Volmer eq 2.^33^

2 $$\frac{F_{0}}{F}=1+K_{sv}\left[Q\right]=1+k_{q}\tau_{0}\left[Q\right]$$

where *F*_0_ is the fluorescence intensity in the absence of the quencher, *F* is the fluorescence intensity in the presence of the quencher, [*Q*] is the quencher concentration and *K*_SV_ is the Stern–Volmer constant, *k*_q_ is the bimolecular quenching constant, τ_0_ is the average lifetime of BSA without the quencher. The constant *K*_sv_ was obtained through a graph of *F*_0_/*F* versus [*Q*] and the constant *k*_q_ was obtained by the ratio between *K*_sv_ and τ_0_ (∼10^–8^ s).

Table 2 shows the values obtained for the constant *K*_sv_, and it can be seen, as the temperature increased, the constant also increased, which indicates the occurrence of a dynamic mechanism. On the other hand, when analyzing the values of *k*_q_, it is observed that the values are all of order 10^13^ M^–1^ s^–1^. These values are greater than the maximum possible for a dynamic mechanism (*k*_q_ = 2.0 × 10^10^ M^–1^ s^–1^), thus indicating that there is also static mechanism involvement. Thus, it can be concluded that the suppression of fluorescence caused by the interaction of ruthenium complexes with BSA occurs both by dynamic and static mechanisms.

**Table 2:** Values of the Constants *K*_sv_, *k*_q_, *K*_b_ and *n* Determined at 298 e 310 K for Complexes **1-4**

complex	T(K)	*K*_sv_ (M^–1^)	*k*_q_ (M^–1^ s^–1^)	*K*_b_ (M^–1^)	*n*
**(1)**	298	(5.24 ± 0.16) × 10^4^	5.24 × 10^13^	(1.44 ± 0.10) × 10^4^	0.89
	310	(5.35 ± 0.31) × 10^4^	5.35 × 10^13^	(1.17 ± 0.16) × 10^4^	0.86
**(2)**	298	(2.53 ± 0.10) × 10^4^	2.53 × 10^13^	(3.68 ± 0.53) × 10^5^	1.23
	310	(2.90 ± 0.09) × 10^4^	2.90 × 10^13^	(8.57 ± 0.27) × 10^6^	1.51
**(3)**	298	(3.77 ± 0.52) × 10^4^	3.77 × 10^13^	(1.10 ± 0.08) × 10^4^	0.90
	310	(4.01 ± 0.70) × 10^4^	4.01 × 10^13^	(1.66 ± 0.12) × 10^4^	0.93
**(4)**	298	(3.53 ± 0.47) × 10^4^	3.53 × 10^13^	(1.20 ± 0.09) × 10^4^	0.88
	310	(3.64 ± 0.91) × 10^4^	3.64 × 10^13^	(2.99 ± 0.71) × 10^5^	1.18

The binding constant (*K*_b_) between the complexes and BSA, as well as the number of binding sites (*n*) were determined using the following eq 3.^33^

3 $$\log[(F0-F)/F]=\log{\;K}_{b}+n\log\left[Q\right]$$

the constant *K*_b_ was obtained from the linear coefficient of the line obtained through the graph of log­(*F*_0_-*F*)/*F* versus log­[*Q*] and *n* was calculated from the slope of this same equation of the line. The *K*_b_ values, shown in Table 2, are in the order of 10^4^–10^6^ M^–1^. These *K*_b_ values show that the interaction strength of the complexes with BSA varies from moderate to strong. Among the complexes, [Ru­(me)­(dppe)_2_]­PF_6_ interacts more strongly with BSA, as it has *K*_b_ of order 10^5^–10^6^ M^–1^. Similar behavior was observed for other ruthenium complexes containing two diphosphines dppe, lapachol, lawsone and 2-mercaptopyrimidine as ligands.^20,34^ With the exception of the complex [Ru­(fe)­(dppe)_2_]­PF_6_, *K*_b_ values for the other complexes increased with rising temperature, thus indicating greater stability of the complex formed with BSA as the temperature increased. The number of binding sites (*n*) for the complexes is approximately 1, indicating that the complexes interact with the protein through one binding site.

The interaction of complexes with BSA can occur through various types of interactions, such as hydrogen bonds, van der Waals forces, hydrophobic or electrostatic interactions. In order to better understand which type of interaction is involved in the evaluated system, thermodynamic parameters such as the Gibbs free energy change (Δ*G*°), enthalpy change (Δ*H*°) and entropy change (Δ*S*°) were determined from the eqs 4 and 5)^35^

4 $$\ln\left(K_{b_{1}}/K_{b_{2}}\right)=(1/T1-{1/T}_{2})\times \Delta H/R$$

5 $$\Delta G^{{}^{\circ}}=-{RT\;\ln K}_{b}=\Delta H^{{}^{\circ}}-T\Delta S^{{}^{\circ}}$$

where *K*_b_1__ and *K*_b_2__ are equivalent to the binding constants in *T*_1_ (298 K) and *T*_2_ (310 K), where *R* is the ideal gas constant.

The obtained values of Δ*G*°, Δ*H*° and Δ*S*° are shown in Table 3 and from the analysis of their signals, it is possible to define the type of interaction involved between the complexes and BSA. Positive values for Δ*H*° and Δ*S*° indicate the involvement of hydrophobic interactions, negative values for Δ*H*° and Δ*S*° correspond to van der Waals strength, hydrogen bonds, negative values for Δ*H*°, and positive for Δ*S*° suggest the involvement of electrostatic interactions. Based on this, the complex [Ru­(fe)­(dppe)_2_]­PF_6_ and BSA interact via electrostatic interactions, and in the case of the other complexes, predominantly hydrophobic interactions are involved. Furthermore, as the values of Δ*G*° are negative, the binding process is spontaneous.

**Table 3:** Gibbs Free Energy Change (Δ*G*°), Enthalpy Change (Δ*H*°) and Entropy Change (Δ*S*°) for Complexes **1-4**

complexes	Δ*G*° (kJ mol^–1^)	Δ*H*° (kJ mol^–1^)	Δ*S*° (J mol^–1^)
(1)	–23.72	–13.29	35.01
	–24.14		33.66
(2)	–31.75	201.49	782.70
	–41.15		752.40
(3)	–23.06	26.34	165.76
	–25.05		159.34
(4)	–23.27	205.82	768.76
	–32.50		739.00

### Cytotoxic Activity of the Complexes **1**-**4**

The cytotoxic activity of ruthenium complexes **1**-**4** was investigated against breast (MDA-MB-231) and lung (A549) tumor cell lines and nontumor lung (MRC-5) cell line, through the MTT assay. The studied cells were treated with the complexes at different concentrations during a period of 48 h. Cisplatin was used as a positive control. IC_50_ values (concentration of compound that inhibits 50% of cell growth) were determined and the results are shown in Table 4. It is noteworthy that the free ligands fe, me, flu and tol were studied separately and did not show cytotoxic activity at the highest concentration used (100 μM). Consequently, it appears that the coordination of fe, me, flu and tol ligands to ruthenium increased their cytotoxic activity, which may be due to the synergistic effect of the metal–ligand combination.

**Table 4:** IC_50_ Values (μM) for Ruthenium and Cisplatin Complexes in MDA-MB-231, A549 and MRC-5 Cell Lines^a^

	IC_50_ (μM)				
	MRC-5	A549	MDA-MB-231	SI^1^	SI^2^
**(1)**	8.40 ± 1.65	2.90 ± 0.11	1.93 ± 0.24	2.90	4.35
**(2)**	7.68 ± 1.65	2.40 ± 0.27	3.67 ± 0.77	3.20	2.09
**(3)**	11.79 ± 2.51	17.71 ± 1.35	6.29 ± 0.28	0.67	1.87
**(4)**	1.98 ± 0.55	1.72 ± 0.09	0.94 ± 0.20	1.15	2.11
*cis-*[RuCl_2_(dppe)_2_]	2.83 ± 0.06	0.40 ± 0.060	0.77 ± 0.40	7.08	3.68
Ligand	>100	>100	>100	--	--
CDDP	29.1 ± 0.8	11.54 ± 1.19	2.43 ± 0.20	2.52	11.98

a Ligand = fe, me, tol and flu, CDDP = Cisplatin. SI = Selectivity Index. *SI^1^ = IC_50_ MRC-5/IC_50_A549 e *SI^2^ = IC_50_ MRC-5/IC_50_MDA-MB-231.

Analyzing the IC_50_ values of the ruthenium complexes, it is verified that all of them demonstrated themselves to be cytotoxic against the investigated cell lines. With the exception of the [Ru­(tol)­(dppe)_2_]­PF_6_ complex, all other complexes, including the precursor *cis*-[RuCl_2_(dppe)_2_],^36^ proved to be more cytotoxic against the A549 cell line than cisplatin, the drug used as a reference. Furthermore, the complexes [Ru­(fe)­(dppe)_2_]­PF_6_ and [Ru­(flu)­(dppe)_2_]­PF_6_ are 1.25 and 2.58 times, respectively, more cytotoxic than cisplatin against the MDA-MB-231 cell line. The [Ru­(me)­(dppe)_2_]­PF_6_ complex showed a selectivity index (SI) of 3.20 for the A549 cell line, compared to the MRC-5 nontumor cell line. In the case of the MDA-MB-231 line, the most selective complex was [Ru­(fe)­(dppe)_2_]­PF_6_, with a selectivity index of 4.35.

One study showed that ruthenium/arene complexes with the mefenamic acid ligand obtained IC_50_ values above 100 μM against the A549 and MDA-MB-231 tumor cell lines.^37^ Another study based on ruthenium complexes and nonsteroidal anti-inflammatory drugs as ligands, including fenamic acid derivatives, indicated IC_50_ values > 30 μM for the A549 cell line.^38^ Thus, we observed that the complexes in this work were very promising against these cell lines, showing higher cytotoxicity.

Other studies containing complexes with the general formula [Ru­(L)­(dppe)_2_]­PF_6_, where L = carboxylic ligand, which indicated high cytotoxicity against the MDA-MB-231 cell line, with IC_50_ in the range of 0.15–3.56 μM, values close to those of the complexes in this work, with the exception of [Ru­(tol)­(dppe)_2_]­PF_6_, reinforcing the importance of the results obtained compared with previously reported data.^20,21,39–41^ Recently, a [Ru­(dppe)_2_]-based complex with 5-fluorouracil has been shown to enhance cytotoxic effects against glioblastoma cells when compared to the free drug, highlighting the potential of this core for bioactive metallodrug design.^42^

A more detailed comparison between the biomolecular interaction studies (ct-DNA and BSA) and the cytotoxicity data reveals a structure–activity relationship among complexes **1**-**4**. Although all compounds present the same [Ru­(L)­(dppe)_2_]^+^ core, their antiproliferative profiles vary markedly depending on the nature of the coordinated NSAID ligand. In this context, NSAID-based metal complexes,^43^ including ruthenium systems, have been reported to enhance anticancer activity through multiple mechanisms, such as biomolecular targeting and modulation of intracellular pathways, thereby supporting the relevance of structure–activity relationship analyses in Ru­(II)/dppe/NSAID derivatives.

Complexes **2** and **3**, which exhibit the highest ct-DNA binding constants (*ca*. 10^5^ M^–1^), also display low micromolar IC_50_ values against the A549 and MDA-MB-231 cell lines, indicating that enhanced ct-DNA affinity contributes to their cytotoxic activity. In contrast, complex **4** shows the most pronounced antiproliferative effect across all tested tumor cell lines despite presenting a ct-DNA binding constant of the same order of magnitude as complex **1**. This finding suggests that only ct-DNA interaction does not fully account for the observed biological activity and that additional factors, such as ligand hydrophobicity and cellular uptake, play a decisive role. This interpretation is further supported by the BSA-binding studies, which reveal stronger and predominantly hydrophobic interactions for complexes **2** and **4**, favoring protein-assisted transport and bioavailability in biological media. Conversely, complex **1**, characterized by weaker interactions with both ct-DNA and BSA, exhibits reduced cytotoxicity and selectivity. Overall, these results indicate that optimal antiproliferative activity is achieved for complexes that combine moderate-to-strong ct-DNA interaction with favorable protein-binding properties, highlighting a synergistic contribution of biomolecular targeting and efficient transport to the overall cytotoxic response.

## Conclusion

In the present report, four new ruthenium­(II)/dppe complexes with fenamate ligands were synthesized and characterized. Spectroscopic and viscosity studies demonstrated that all complexes interact moderately with ct-DNA. The interaction of the complexes with bovine serum albumin (BSA) showed that the compound [Ru­(fe)­(dppe)_2_]­PF_6_ demonstrated probable electrostatic interactions, while the other compounds probably have hydrophobic interactions with BSA. Finally, all the studied compounds showed good IC_50_ values when compared to cisplatin. By the selectivity index (SI), the compounds [Ru­(fe)­(dppe)_2_]­PF_6_ and [Ru­(me)­(dppe)_2_]­PF_6_ presented the best results in A549 cells. For the MDA-MB-231 cell, the compound that presented the best SI was [Ru­(fe)­(dppe)_2_]­PF_6_. Importantly, comparison of the biomolecular interaction data with the cytotoxicity results highlights a structure–activity relationship. Although all complexes share the same {Ru­(dppe)_2_} core, variations in the coordinated fenamic ligands significantly influence ct-DNA affinity, protein binding behavior, and antiproliferative activity. Complexes displaying a favorable balance between moderate DNA binding and effective BSA interaction exhibited lower IC_50_ values and higher selectivity toward tumor cell lines. In contrast, the complexes **3** and **4** with ligands presenting halogenated substituents showed reduced selectivity indices, indicating that the presence of halogen substitution in the ligand does not enhance the selectivity of this class of compounds. Overall, these results demonstrate that the biological activity of ruthenium­(II)/dppe/fenamic complexes is governed by a synergistic interplay between complex structure, biomolecular interactions, and cytotoxic response, emphasizing the importance of NSAID ligand for the development of ruthenium-based complexes as anticancer agents.

## Supplementary Material











## Notes

FigureS S1–S4 (Absorption spectrum in the infrared region for complexes, ligands and precursor). Figure S5–S9 (UV–vis spectra of complexes **1-4**). Figure S10–S21 (^1^H, ^13^C and ^31^P NMR spectra for complexes **1-4**). Figure S22–S25 (Cyclic voltammetry of complexes **1-4**). Tables S1–S4 (X-ray data collections and refinement details of complexes **1-4**) (PDF)
CCDC 2388262 (CIF)
CCDC 2388263 (CIF)
CCDC 2388264 (CIF)
CCDC 2388265 (CIF)
