# Supplementary material for: Synthesis and Structure Elucidation of New NSAID Diphosphine Ruthenium(II) Complexes as Potential Anticancer Agents: DNA/BSA Binding and Cytotoxicity Assays
Source: ACS Omega. 2026 Apr 8;11(15):23079–89. doi: 10.1021/acsomega.5c12981 (PMC13103785; doi:10.1021/acsomega.5c12981)
Supplement: Supplementary file 1 [file ao5c12981_si_001.pdf]

# Supporting Information

## Synthesis and structure elucidation of new NSAID diphosphine ruthenium(II) complexes as potential anticancer agents: DNA/BSA binding and cytotoxicity assays

Diogo E. L. Carvalho[a], Tamara Teixeira[a], João Honorato de Araujo-Neto[b], Alzir A. Batista[b], Keven S. Fragoso[c], Katia M. Oliveira[c*], Rodrigo S. Corrêa[a*]

[a] Department of Chemistry, Federal University of Ouro Preto (UFOP), zip code 35402-136, Ouro Preto, Minas Gerais, Brazil;

[b] Department of Chemistry, Federal University of São Carlos (UFSCar), zip code 13561-901, São Carlos, SP, Brazil;

[c] Institute of Chemistry, University of Brasília (UnB), Campus Darcy Ribeiro, zip code 70910-900, Brasília, DF, Brazil.

* *Corresponding authors:*

Kátia M. Oliveira (e-mail: katia.oliveira@unb.br) and Rodrigo S. Corrêa (e-mail: rodrigocorrea@ufop.edu.br)

# IR spectra

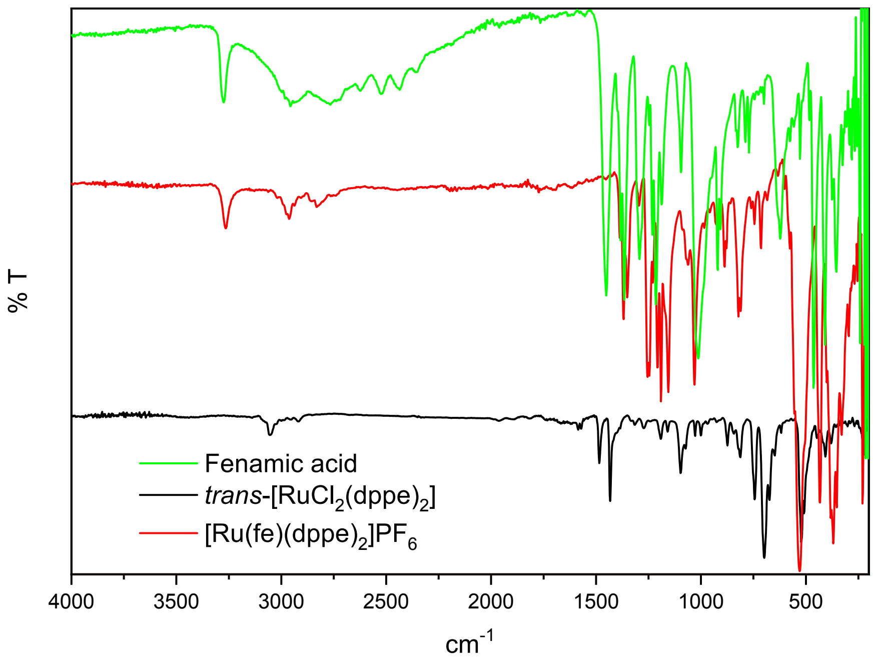


Figure S1 - Absorption spectrum in the infrared region (FTIR-ATR) for the $[Ru(fe)(dppe)_2]PF_6$ (**1**), free ligand and the precursor complex.

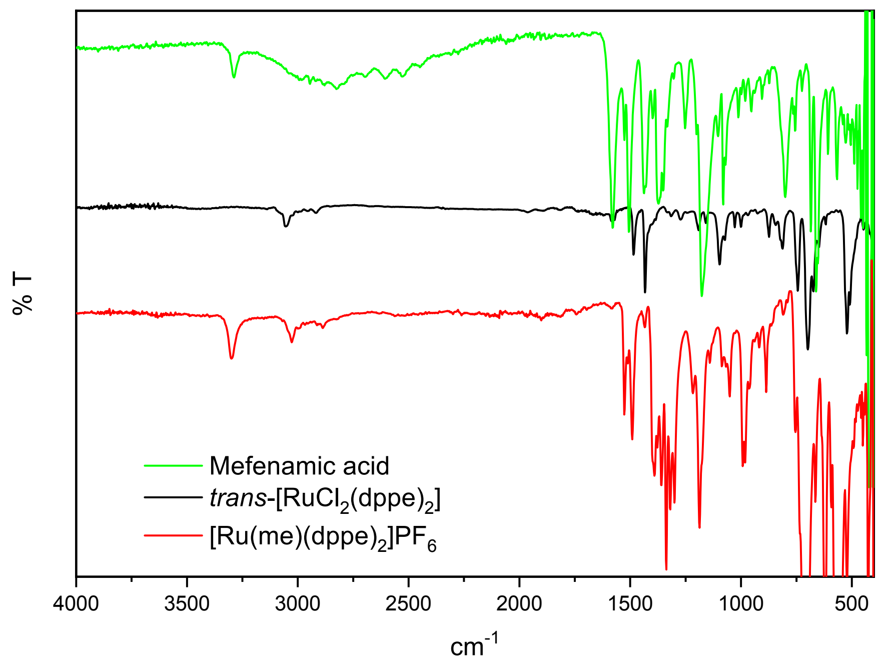


Figure S2 - Absorption spectrum in the infrared region (FTIR-ATR) for the $[Ru(me)(dppe)_2]PF_6$ (**2**), free ligand and the precursor complex.

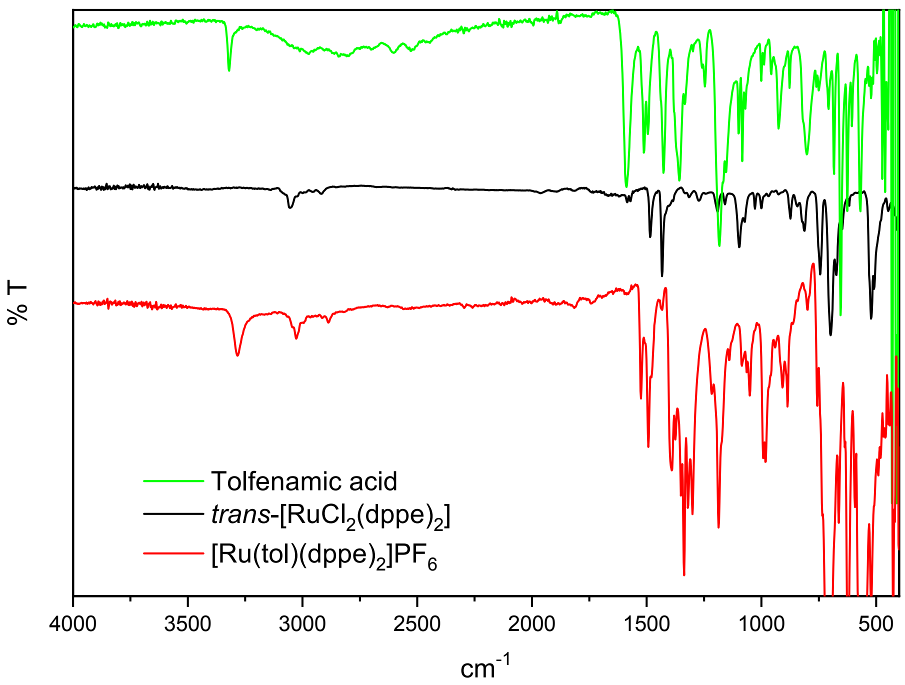


Figure S3 - Absorption spectrum in the infrared region (FTIR-ATR) for the $[Ru(tol)(dppe)_2]PF_6$ (**3**), free ligand and the precursor complex.

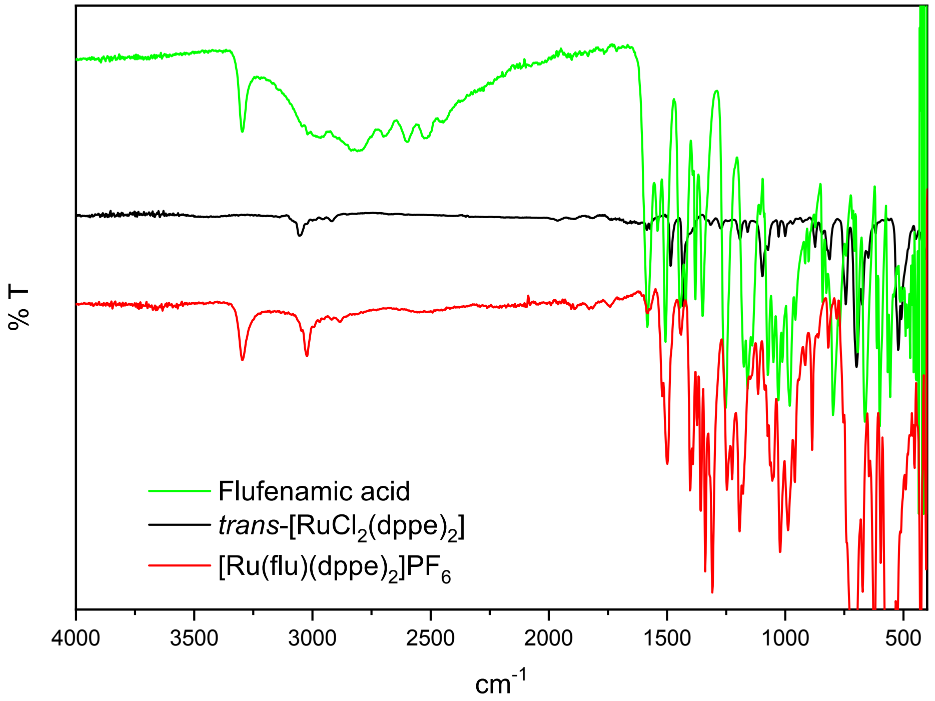


Figure S4 - Absorption spectrum in the infrared region (FTIR-ATR) for the $[Ru(flu)(dppe)_2]PF_6$ (**4**), free ligand and the precursor complex.

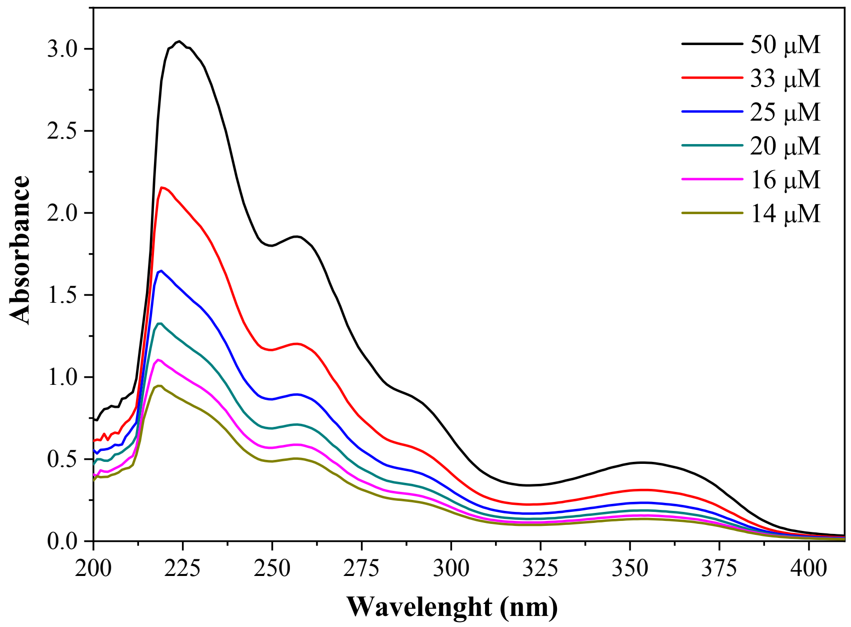


Figure S5 - Absorption spectrum in the ultraviolet and visible region for the $[Ru(fe)(dppe)_2]PF_6$, in methanol.

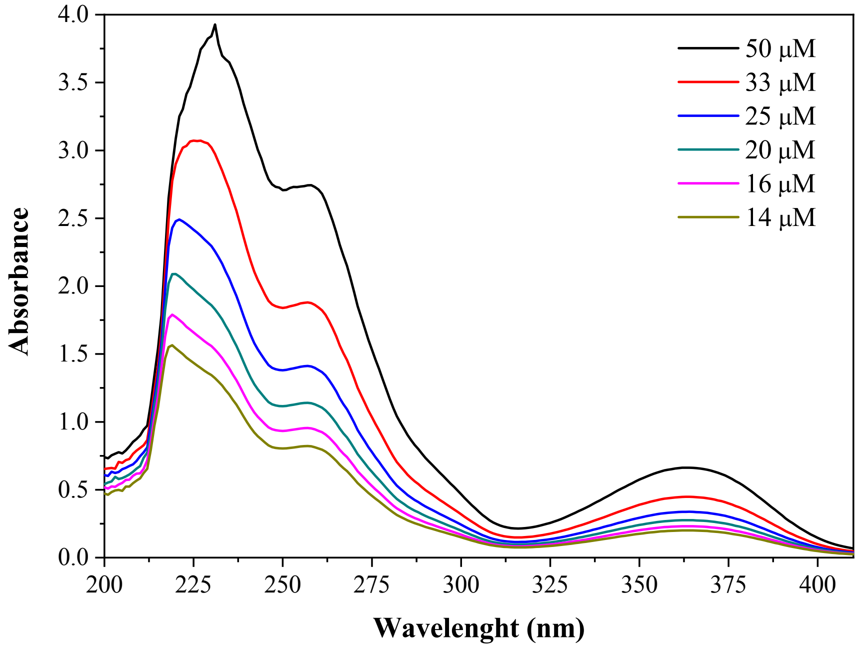


Figure S6 - Absorption spectrum in the ultraviolet and visible region for the complex $[Ru(me)(dppe)_2]PF_6$, in methanol.

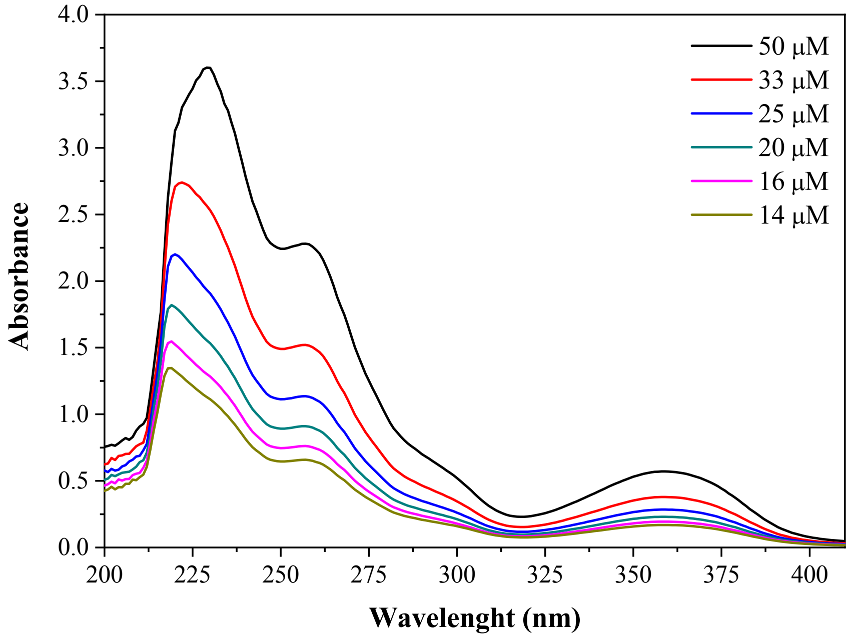


Figure S7 - Absorption spectrum in the ultraviolet and visible region for the complex $[Ru(tol)(dppe)_2]PF_6$, in methanol.

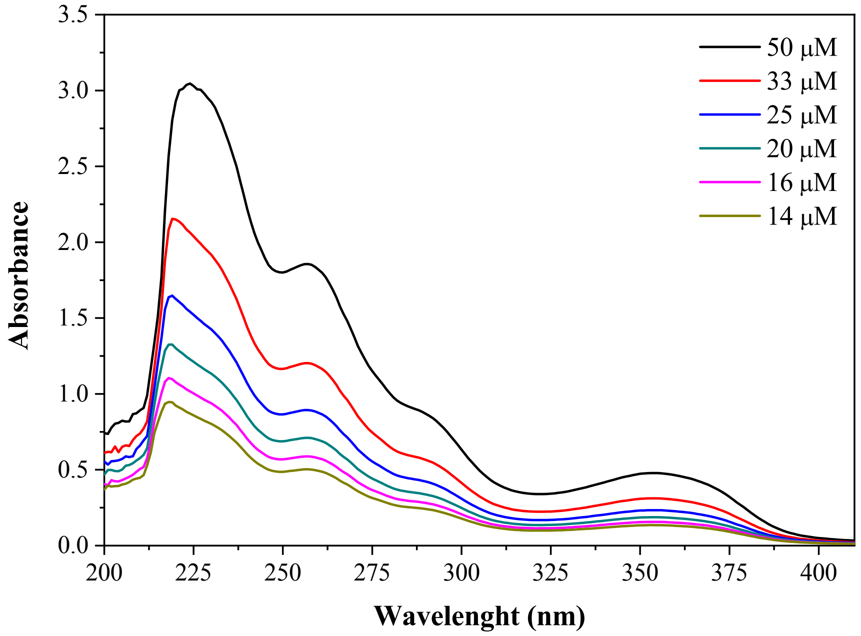


Figure S8 - Absorption spectrum in the ultraviolet and visible region for the complex [Ru(flu)(dppe)$_2$]PF$_6$, in methanol.

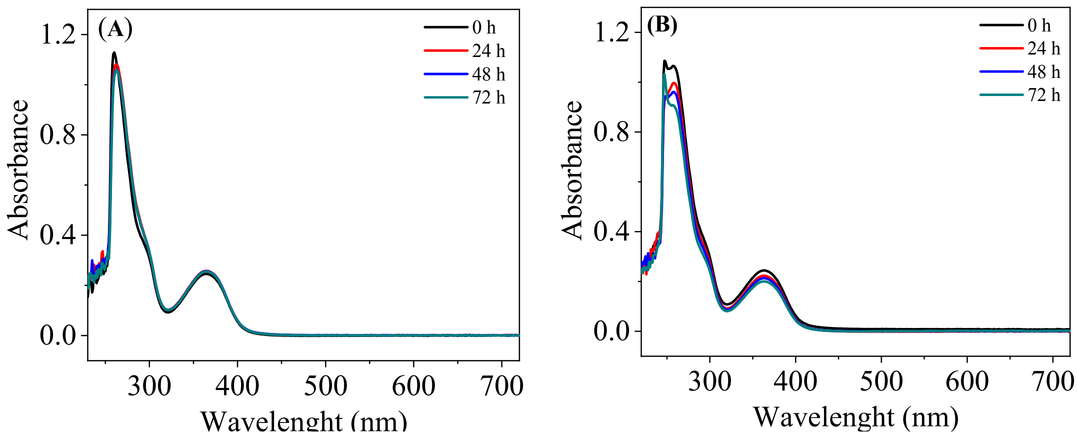


Figure S9 - Absorption spectra in the UV-vis region of the [Ru(fe)(dppe)$_2$]PF$_6$ complex in (A) DMSO and (B) DMSO/Tris-HCl Buffer 50:50 (v/v).

# NMR spectra

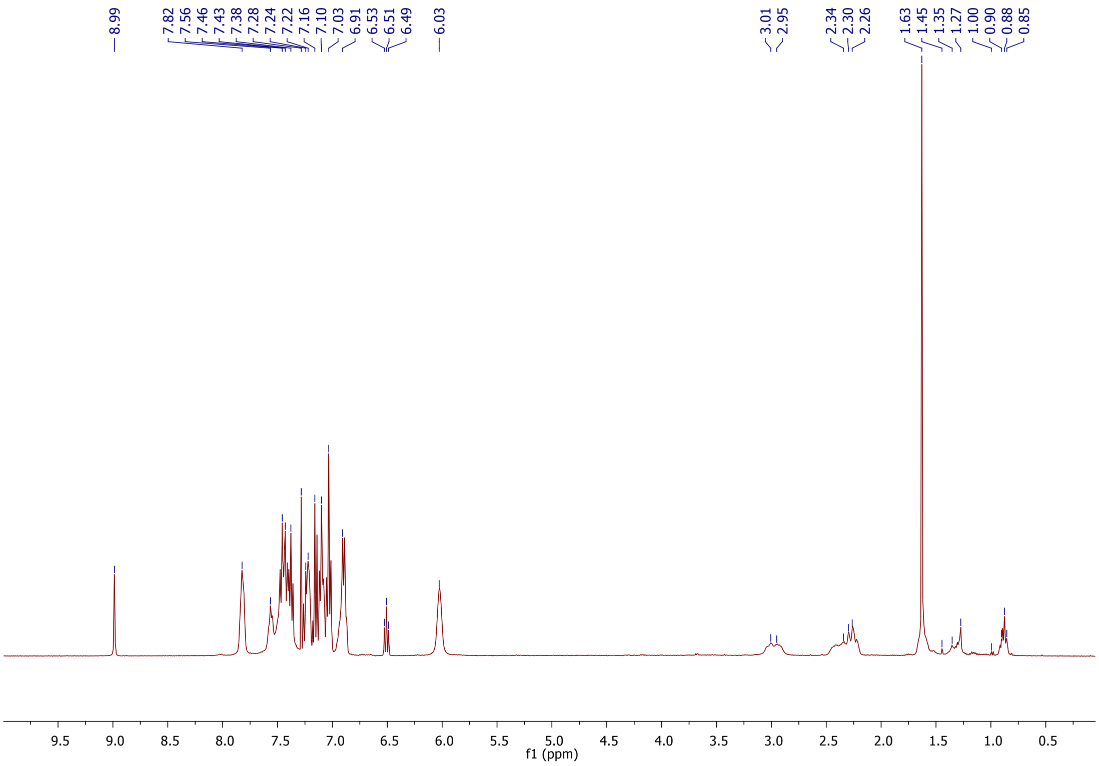


Figure S10 - $^{1}$H NMR of [Ru(fe)(dppe)$_2$]PF$_6$ in CDCl$_3$.

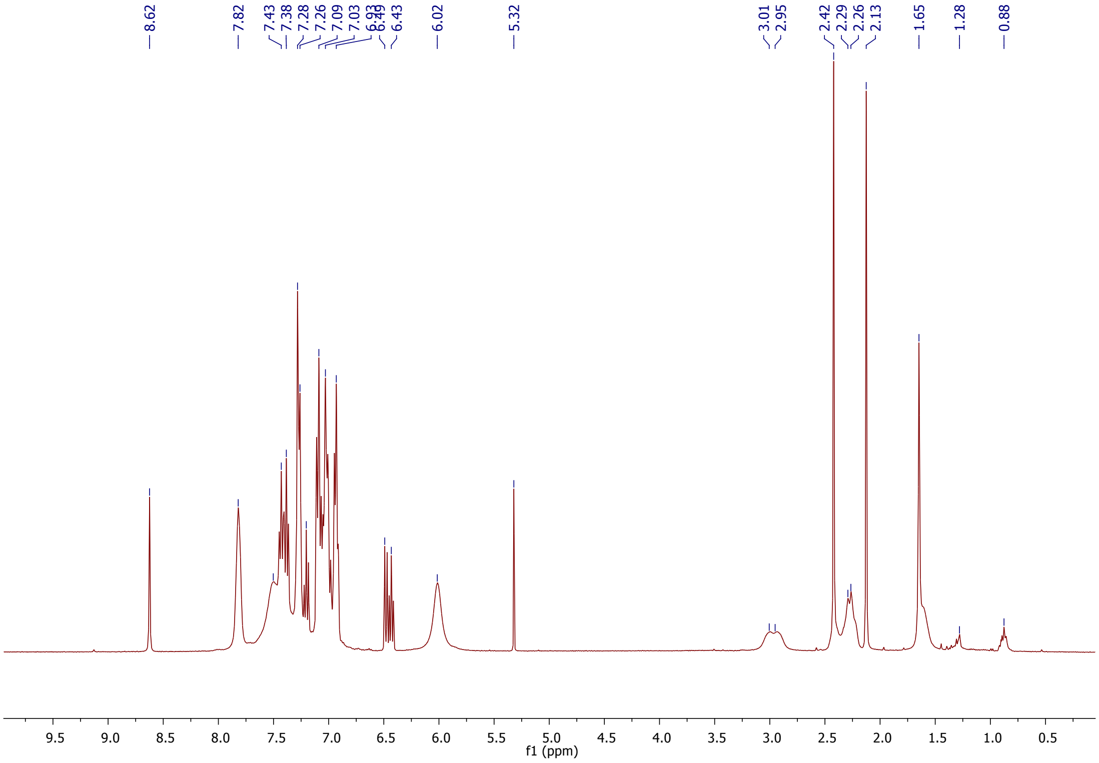


Figure S11 - $^{1}$H NMR of [Ru(me)(dppe)$_2$]PF$_6$ in CDCl$_3$.

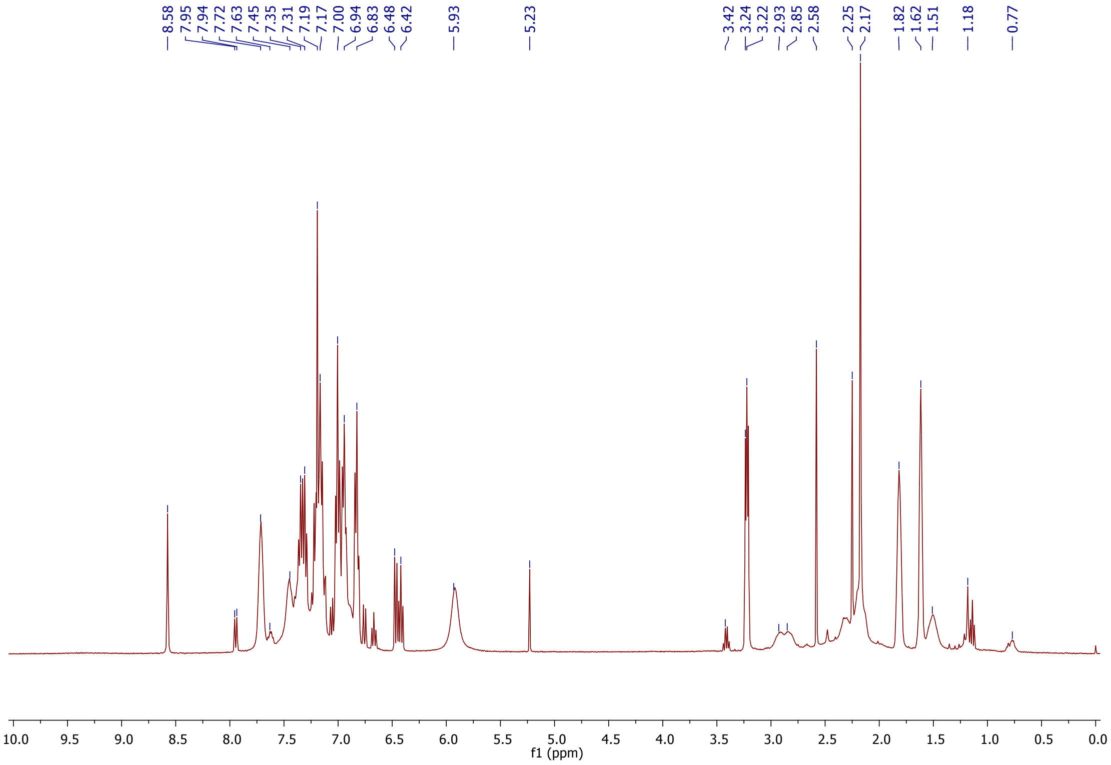


Figure S12 - $^{1}H$ NMR of $[Ru(tol)(dppe)_2]PF_6$ in $CDCl_3$.

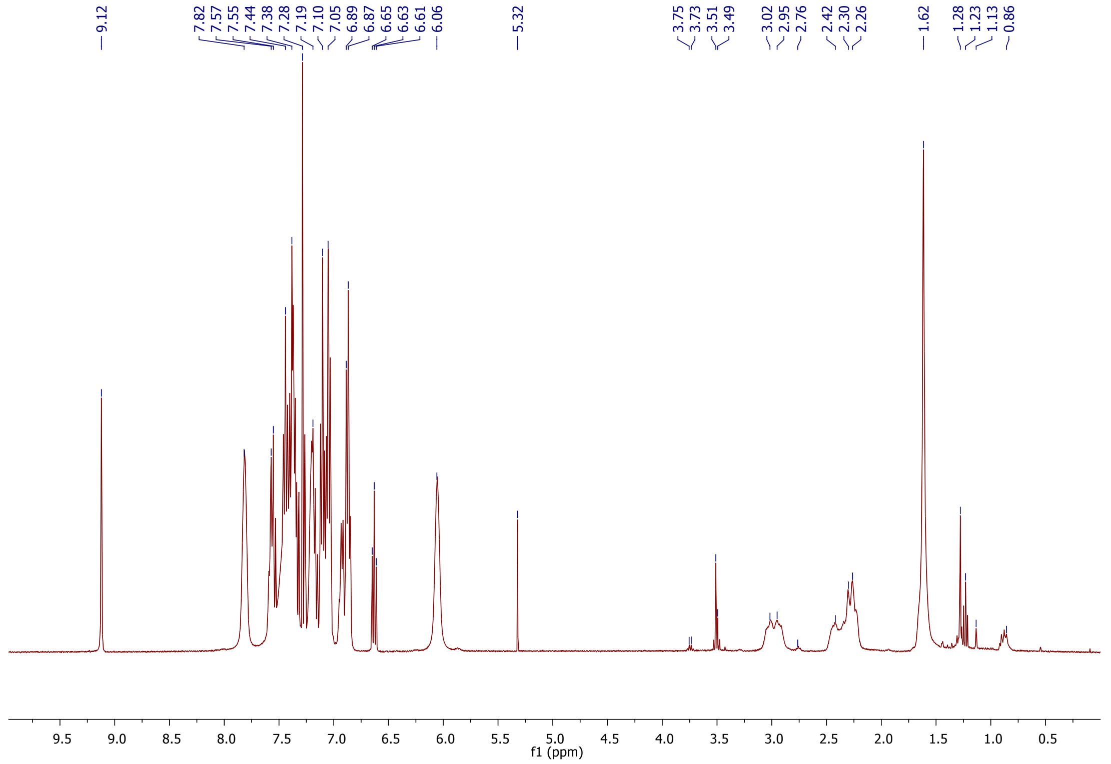


Figure S13 - $^{1}H$ NMR of $[Ru(flu)(dppe)_2]PF_6$ in $CDCl_3$.

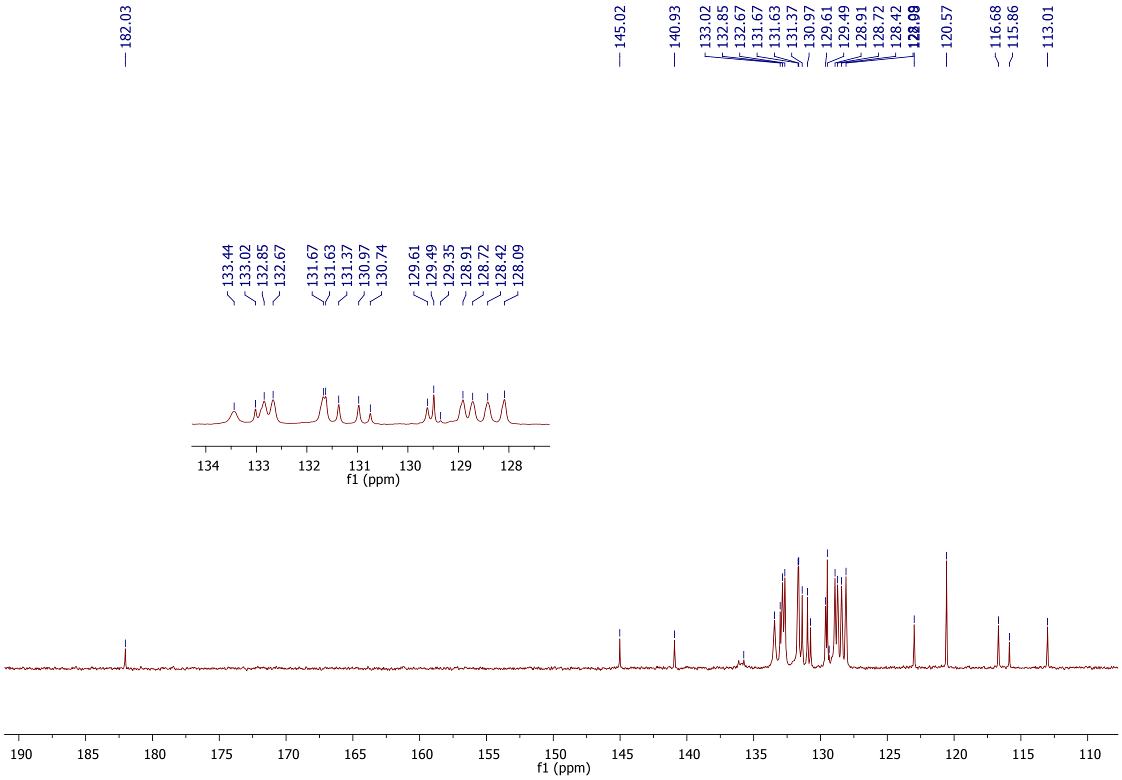

Figure S14 - $^{13}C\{^1H\}$ NMR of $[Ru(fe)(dppe)_2]PF_6$ in $CDCl_3$.

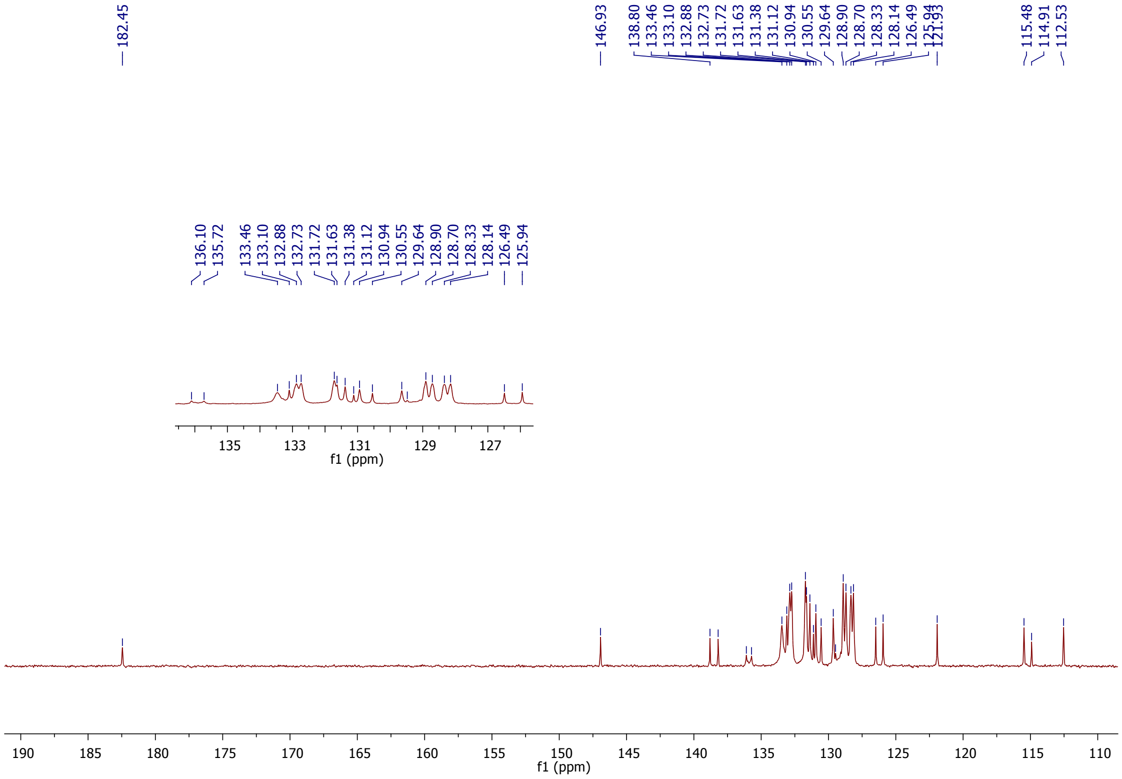

Figure S15 - $^{13}C\{^1H\}$ NMR of $[Ru(me)(dppe)_2]PF_6$ in $CDCl_3$.

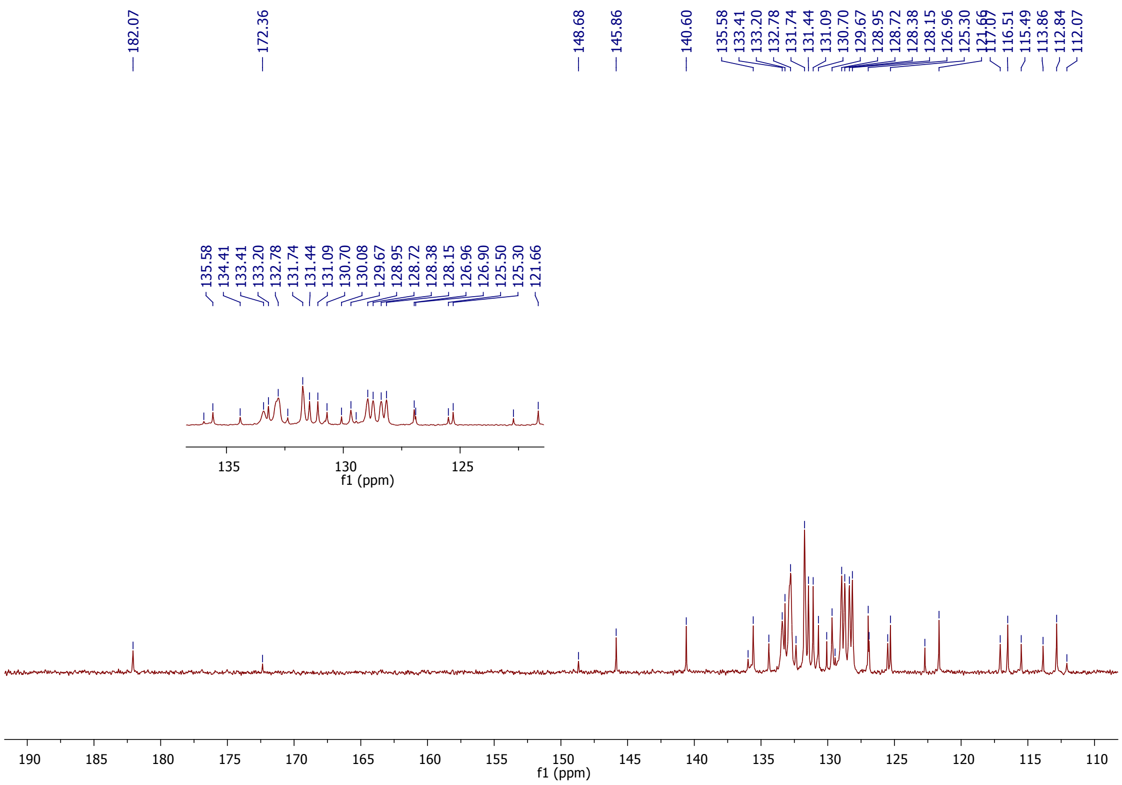


Figure S16 - $^{13}C\{^{1}H\}$ NMR of $[Ru(tol)(dppe)_2]PF_6$ in $CDCl_3$.

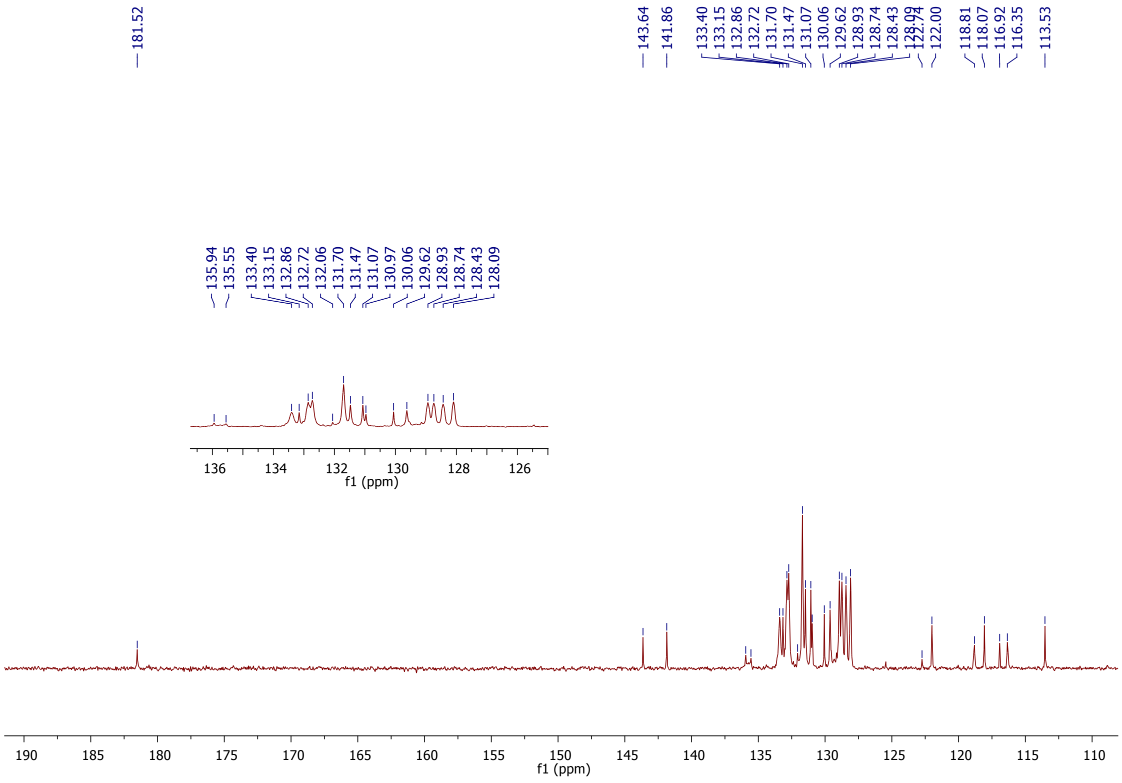


Figure S17 - $^{13}C\{^{1}H\}$ NMR of $[Ru(flu)(dppe)_2]PF_6$ in $CDCl_3$.

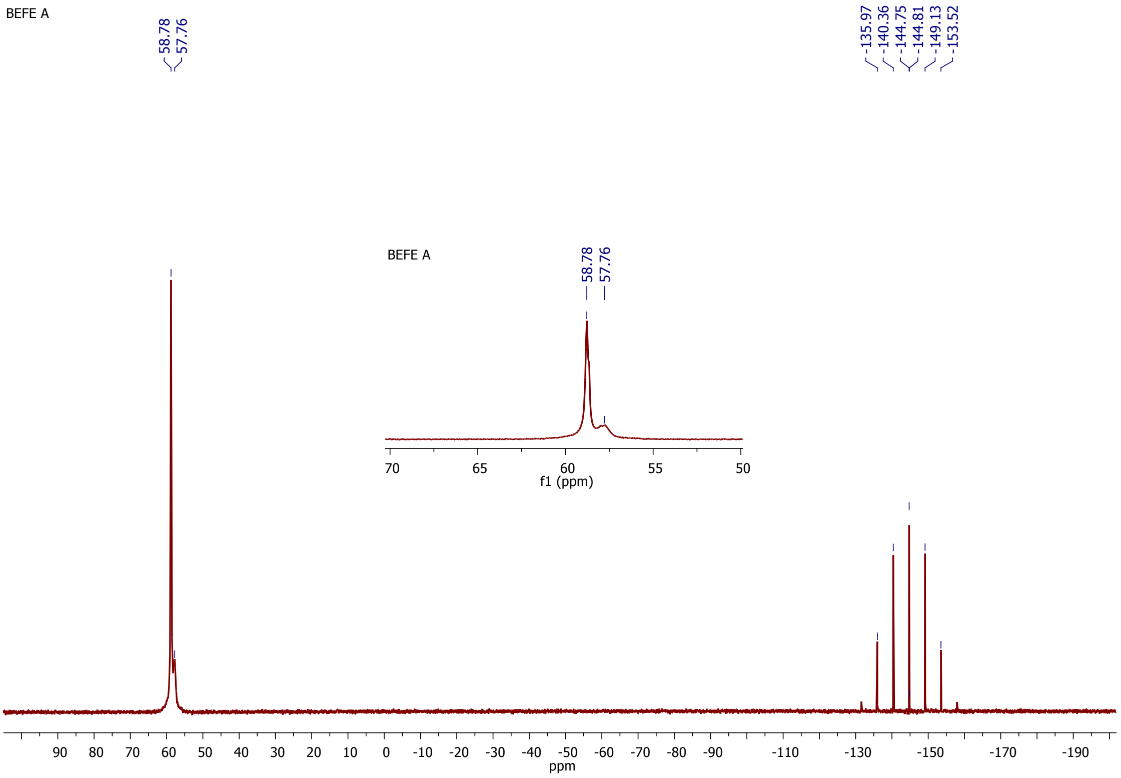


Figure S18 – $^{31}P\{^{1}H\}$ NMR of $[Ru(fe)(dppe)_2]PF_6$ in $CDCl_3$.

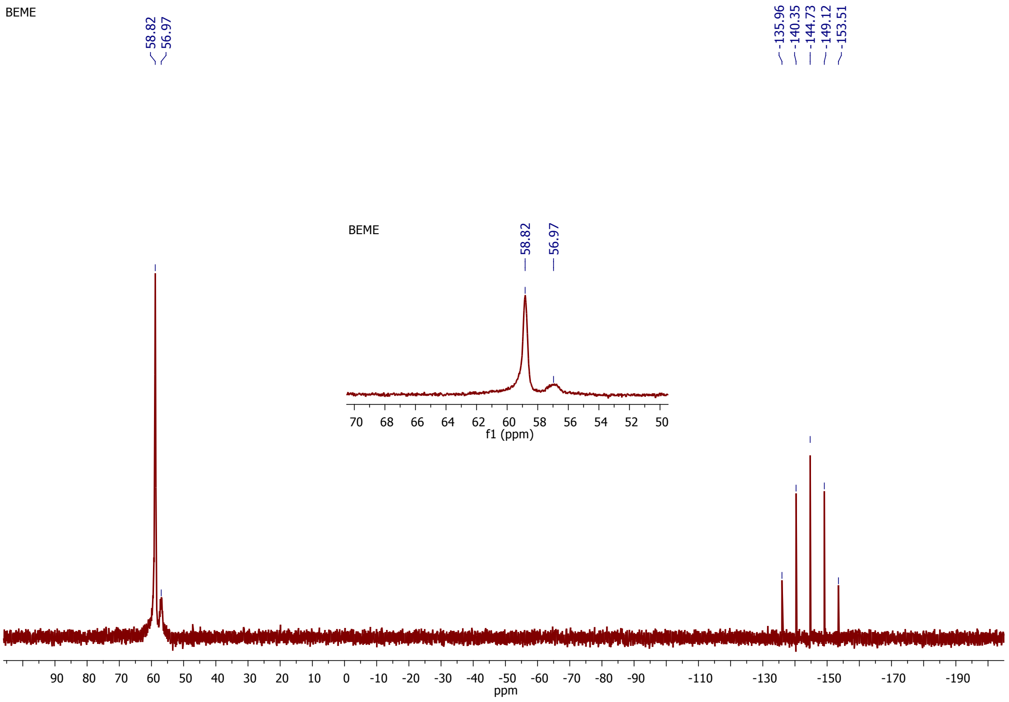


Figure S19 – $^{31}P\{^{1}H\}$ NMR of $[Ru(me)(dppe)_2]PF_6$ in $CDCl_3$.

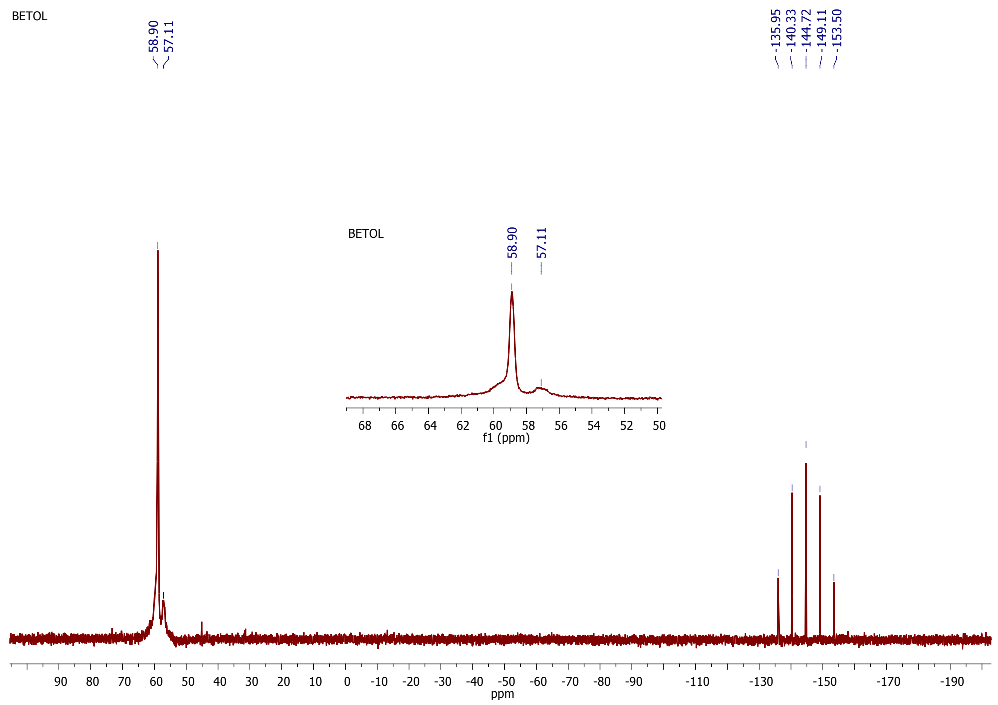


Figure S20 – $^{31}P\{^{1}H\}$ NMR of $[Ru(tol)(dppe)_2]PF_6$ in $CDCl_3$.

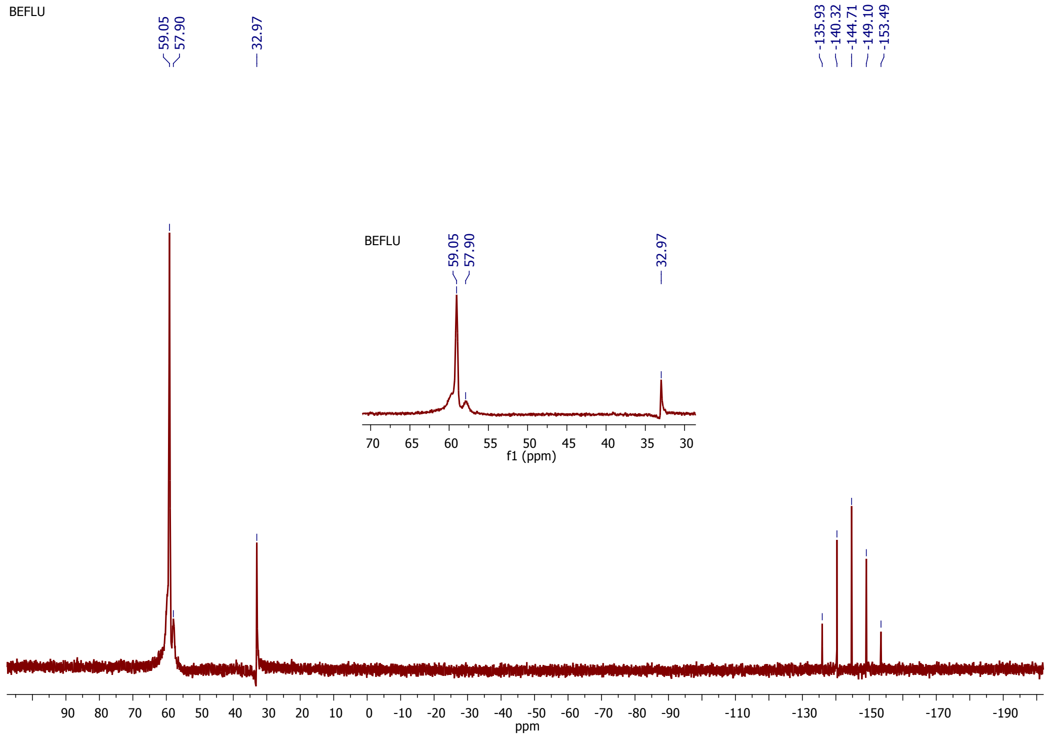


Figure S21 – $^{31}P\{^{1}H\}$ NMR of $[Ru(flu)(dppe)_2]PF_6$ in $CDCl_3$.

# Electrochemical data

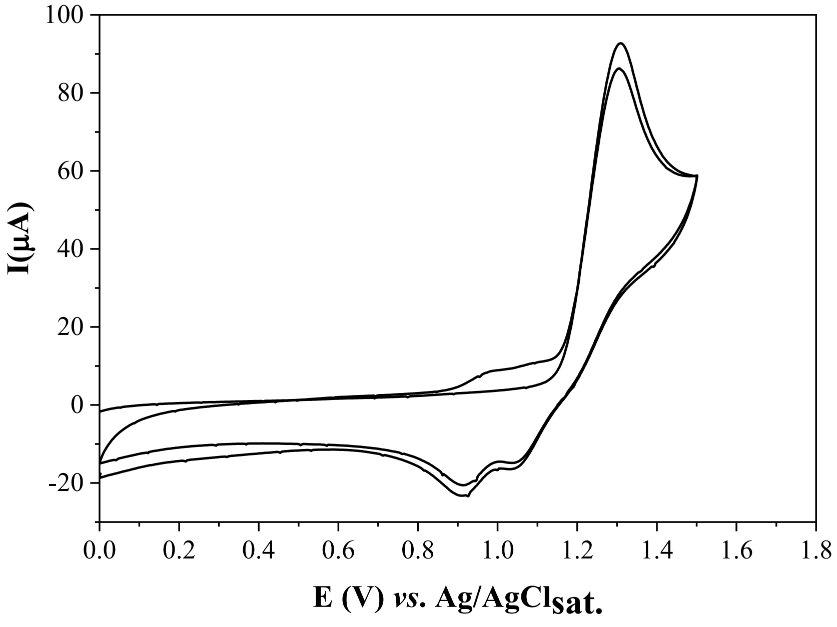


Figure S22 - Cyclic voltammogram for the complex $[Ru(fe)(dppe)_2]PF_6$ in $CH_2Cl_2$ ($1x10^{-3}$ molL$^{-1}$ of PTBA).

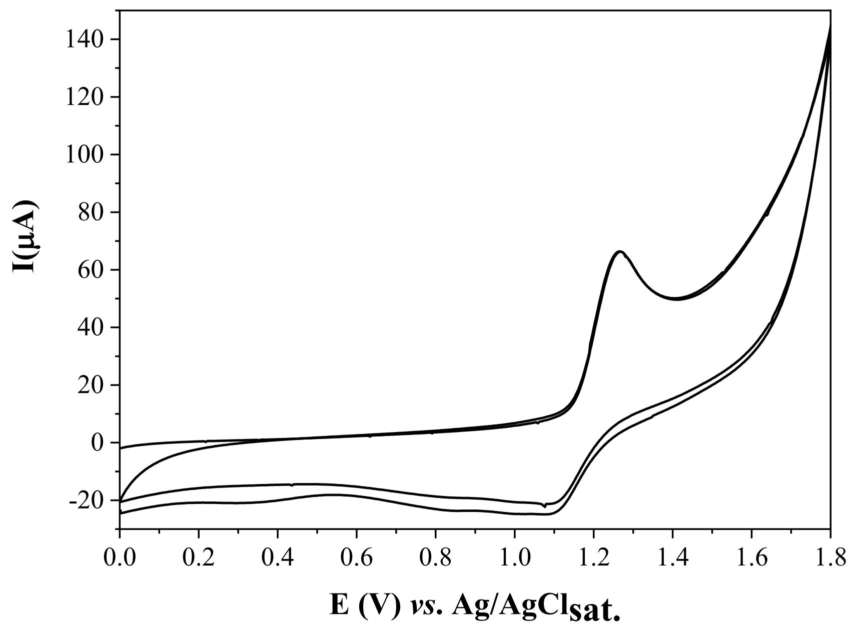


Figure S23 - Cyclic voltammogram for the complex $[Ru(me)(dppe)_2]PF_6$ in $CH_2Cl_2$ ($1x10^{-3}$ $molL^{-1}$ of PTBA).

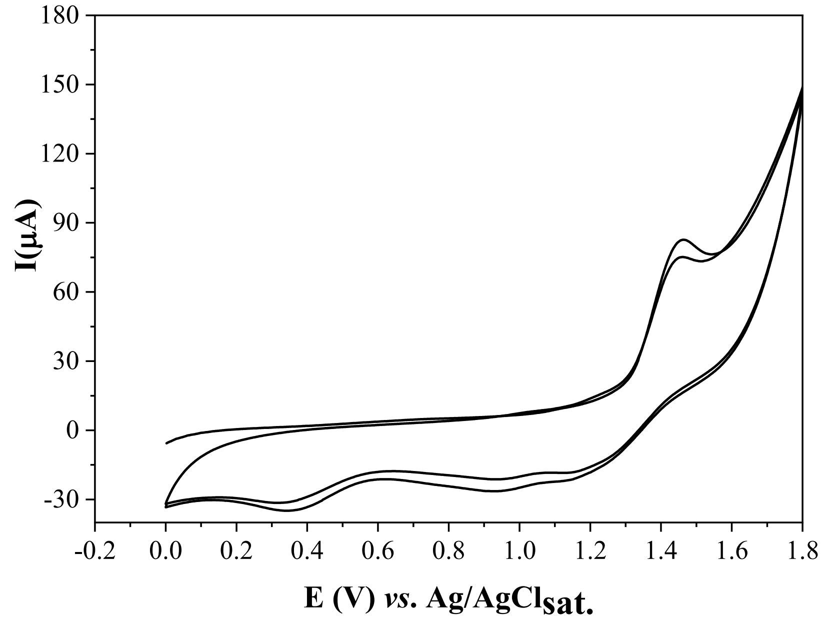


Figure S24 - Cyclic voltammogram for the complex $[Ru(tol)(dppe)_2]PF_6$ in $CH_2Cl_2$ ($1x10^{-3}$ $molL^{-1}$ of PTBA).

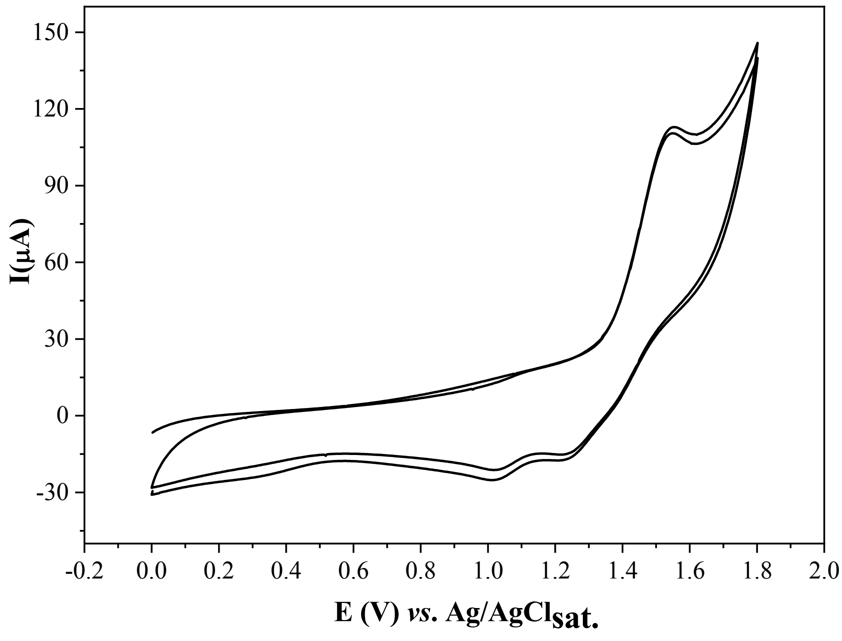


Figure S25 - Cyclic voltammogram for the complex [Ru(flu)(dppe)$_2$]PF$_6$ in $CH_2Cl_2$ (1x10$^{-3}$ molL$^{-1}$ of PTBA).

# Crystallographic data and details of the refinement for 1-4

Table 1S. Crystal data and structure refinement for **1**.

| | | |
|---|---|---|
| Empirical formula | $C_{65}H_{58}$ $F_6$ N $O_2$ $P_5$ Ru | |
| Formula weight | 1255.04 | |
| Temperature | 293(2) K | |
| Wavelength | 0.71073 Å | |
| Crystal system | Monoclinic | |
| Space group | C 2/c | |
| Unit cell dimensions | a = 25.7097(6) Å | |
| | b = 12.1820(2) Å | β = 101.272(2)°. |

| | |
|---|---|
| | c = 37.2901(9) Å |
| Volume | 11453.8(4) $Å^3$ |
| Z | 8 |
| Density (calculated) | 1.456 $Mg/m^3$ |
| Absorption coefficient | 0.480 $mm^{-1}$ |
| F(000) | 5152 |
| Crystal size | 0.30 x 0.12 x 0.08 $mm^3$ |
| Theta range for data collection | 2.60 to 26.00°. |
| Index ranges | -31≤h≤31, -15≤k≤15, -45≤l≤45 |
| Reflections collected | 57154 |
| Independent reflections | 11258 [R(int) = 0.0453] |
| Completeness to theta = 25.242° | 99.9 % |
| Refinement method | Full-matrix least-squares on $F^2$ |
| Data / restraints / parameters | 11258 / 0 / 724 |
| Goodness-of-fit on $F^2$ | 1.229 |
| Final R indices [I>2sigma(I)] | R1 = 0.0687, wR2 = 0.1212 |
| R indices (all data) | R1 = 0.0789, wR2 = 0.1272 |
| Extinction coefficient | n/a |
| Largest diff. peak and hole | 0.704 and -1.287 e.$Å^{-3}$ |

Table 2S. Crystal data and structure refinement for **2**.

| | | |
|---|---|---|
| Empirical formula | $C_{67} H_{62} F_6 N O_2 P_5 Ru$ | |
| Formula weight | 1283.09 | |
| Temperature | 293(2) K | |
| Wavelength | 0.71073 Å | |
| Crystal system | Monoclinic | |
| Space group | Cc | |
| Unit cell dimensions | a = 19.6292(15) Å | |
| | b = 15.9250(11) Å | β = 105.090(8)°. |
| | c = 19.2447(16) Å | |
| Volume | 5808.4(8) $Å^3$ | |

| | |
|---|---|
| Z | 4 |
| Density (calculated) | 1.467 Mg/m$^3$ |
| Absorption coefficient | 0.475 mm$^{-1}$ |
| F(000) | 2640 |
| Crystal size | 0.240 x 0.148 x 0.101 mm$^3$ |
| Theta range for data collection | 2.524 to 25.999°. |
| Index ranges | -24≤h≤24, -19≤k≤19, -23≤l≤23 |
| Reflections collected | 58152 |
| Independent reflections | 11405 [R(int) = 0.0498] |
| Completeness to theta = 25.242° | 99.9 % |
| Refinement method | Full-matrix least-squares on F$^2$ |
| Data / restraints / parameters | 11405 / 2 / 741 |
| Goodness-of-fit on F$^2$ | 1.095 |
| Final R indices [I>2sigma(I)] | R1 = 0.0358, wR2 = 0.0807 |
| R indices (all data) | R1 = 0.0506, wR2 = 0.0901 |
| Absolute structure parameter | 0.94(4) |
| Largest diff. peak and hole | 0.460 and -0.334 e.Å$^{-3}$ |

Table 3S. Crystal data and structure refinement for **3**.

| | | |
|---|---|---|
| Empirical formula | $C_{66}H_{59}ClF_6NO_2P_5Ru$ | |
| Formula weight | 1303.51 | |
| Temperature | 293(2) K | |
| Wavelength | 0.71073 Å | |
| Crystal system | Monoclinic | |
| Space group | Cc | |
| Unit cell dimensions | a = 19.6387(7) Å | |
| | b = 15.8829(5) Å | β = 105.508(4)°. |
| | c = 19.2176(7) Å | |
| Volume | 5776.1(4) Å$^3$ | |
| Z | 4 | |
| Density (calculated) | 1.499 Mg/m$^3$ | |

| | |
|---|---|
| Absorption coefficient | 0.523 $mm^{-1}$ |
| F(000) | 2672 |
| Crystal size | 0.25 x 0.18 x 0.05 $mm^3$ |
| Theta range for data collection | 2.635 to 25.748°. |
| Index ranges | -24≤h≤23, -19≤k≤19, -23≤l≤23 |
| Reflections collected | 29499 |
| Independent reflections | 10863 [R(int) = 0.0237] |
| Completeness to theta = 25.242° | 99.9 % |
| Refinement method | Full-matrix least-squares on $F^2$ |
| Data / restraints / parameters | 10863 / 2 / 739 |
| Goodness-of-fit on $F^2$ | 1.115 |
| Final R indices [I>2sigma(I)] | R1 = 0.0380, wR2 = 0.0938 |
| R indices (all data) | R1 = 0.0488, wR2 = 0.1037 |
| Absolute structure parameter | 0.31(2) |
| Largest diff. peak and hole | 0.425 and -0.354 e.$Å^{-3}$ |

Table 4S. Crystal data and structure refinement for **4**.

| | | |
|---|---|---|
| Empirical formula | $C_{66}$ $H_{57}F_9NO_2P_5Ru$ | |
| Formula weight | 1323.04 | |
| Temperature | 293(2) K | |
| Wavelength | 0.71073 Å | |
| Crystal system | Monoclinic | |
| Space group | C c | |
| Unit cell dimensions | a = 19.5508(6) Å | |
| | b = 15.8677(5) Å | β = 99.713(3)°. |
| | c = 19.2157(7) Å | |
| Volume | 5875.8(3) $Å^3$ | |
| Z | 4 | |
| Density (calculated) | 1.496 $Mg/m^3$ | |
| Absorption coefficient | 0.479 $mm^{-1}$ | |

| | |
|---|---|
| F(000) | 2704 |
| Crystal size | 0.36 x 0.15 x 0.10 $mm^3$ |
| Theta range for data collection | 2.567 to 26.999°. |
| Index ranges | -24≤h≤24, -18≤k≤20, -24≤l≤24 |
| Reflections collected | 26808 |
| Independent reflections | 12514 [R(int) = 0.0326] |
| Completeness to theta = 25.242° | 99.9 % |
| Refinement method | Full-matrix least-squares on $F^2$ |
| Data / restraints / parameters | 12514 / 2 / 793 |
| Goodness-of-fit on $F^2$ | 1.029 |
| Final R indices [I>2sigma(I)] | R1 = 0.0341, wR2 = 0.0794 |
| R indices (all data) | R1 = 0.0506, wR2 = 0.0879 |
| Absolute structure parameter | -0.01(4) |
| Largest diff. peak and hole | 0.313 and -0.521 e.$Å^{-3}$ |
